# Qualification of a Swellable Hydrogel–Silicone Bimorph for Implantable Actuation

**DOI:** 10.3390/polym18192437

**Published:** 2026-10-07

**Authors:** Esma Dosdoğru, Adrian Onken, Sabine Hild, Patricia Torgau, David Schäffl, Jan Sündermann, Marc Müller, Johannes Aucktor, Hasso v. Zychlinski, Theodor Doll

**Affiliations:** 1 Department of Otorhinolaryngology, Hannover Medical School, Carl-Neuberg-Straße 1, 30625 Hannover, Germany; torgau.patricia@mh-hannover.de (P.T.); doll.theodor@m-hannover.de (T.D.); 2 Institute for Multiphase Processes (IMP), Leibniz University Hannover, An der Universität 1, 30823 Garbsen, Germany; mueller@imp.uni-hannover.de; 3 Institute for Polymer Sciences, Johannes Kepler University Linz, Altenbergerstrasse 69, 4040 Linz, Upper Austria, Austria; sabine.hild@jku.at (S.H.); david.schaeffle@jku.at (D.S.); 4 Fraunhofer Institute for Toxicology and Experimental Medicine ITEM, Nikolai-Fuchs-Straße 1, 30625 Hannover, Germany; jan.suendermann@item.fraunhofer.de; 5 Unavera Chemlab, Am Ländbach 20, 82481 Mittenwald, Germany; johannes@unavera.de (J.A.); hasso@unavera.de (H.v.Z.)

**Keywords:** silicone–hydrogel hybrids, cytotoxicity, stimuli-responsive hydrogels, swelling behavior, implantable medical devices, cochlear implants

## Abstract

To improve the structural adaptability and biological suitability of cochlear implant electrode carriers, this study systematically develops and evaluates novel 3D-printed, swellable hydrogel–silicone composites. A vinylphosphonic acid–acrylamide hydrogel powder (VPA-AAm) was pre-washed, milled, and sieved, incorporated into a silicone matrix, and 3D-printed before rapid thermal curing, followed by microstructural, swelling, and in vitro WST-1 cytotoxicity analyses. Pre-washing minimized particle agglomeration and facilitated high-resolution printing. Incorporating the hydrogel induced complete macroscopic opacity at 25 wt.% and localized microporosity during rapid thermal curing. The non-percolating morphology influenced swelling and mass transport through the composite, providing a mechanistic link between water uptake and the observed cell-viability response. While a 35 wt.% loading was associated with matrix degradation and cell viability below the 70% threshold, the 30 wt.% composition achieved the highest water absorption of approximately 400–460%, consistent with a favorable balance between osmotic swelling and matrix elasticity. This swelling behavior was associated with controlled release characteristics and non-cytotoxic extract responses at intermediate hydrogel loading. Consequently, the 30 wt.% composite represents a promising material combination for implantable medical devices, balancing swelling performance, structural stability, and favorable in vitro cell viability under the investigated extraction conditions.

## 1. Introduction

Stimuli-responsive polymeric materials have attracted increasing attention in recent years due to their potential to enable adaptive, soft, and bioinspired systems. Among these materials, swellable hydrogels are of particular interest, as they can undergo pronounced and reversible volume changes in response to environmental stimuli such as solvent uptake, temperature, or pH variations [1,2,3,4]. When combined with non-swellable elastomeric carrier materials, swellable hydrogels can create hybrid systems, hereafter referred to as bimorphs, capable of converting volumetric swelling into controlled deformation, force generation, or mechanical work [2,5,6,7]. The constrained swelling of the hydrogel layer induces bending or shape changes in the overall structure [6,8,9,10]. Silicone elastomers are widely used in medical technology and already constitute the established carrier material of cochlear implant electrode arrays [8,11]. Their mechanical flexibility, chemical stability, and established use in implantable devices make them particularly attractive for the present concept, as the passive actuator can be developed on an existing material platform rather than requiring the introduction of an entirely new structural carrier material. The NuSil MED-4850 used in this study belongs to the manufacturer’s Implant Line and may be considered for human implantation for periods exceeding 29 days [12]. For such applications, swelling-induced bending provides a potential mechanism for passive adaptation of the electrode carrier to the curved intracochlear anatomy, bringing electrode contacts closer to target neural structures [8]. Building on this concept, the present study focuses on the material and processing requirements of the swellable composite rather than on demonstrating the complete electrode carrier.

Despite the growing body of research on hydrogel-based actuators, significant uncertainties remain regarding the reproducibility and reliability of hydrogel–silicone bimorphs. Previous studies have reported pronounced scatter in swelling behavior as well as inconsistent results in cell-based cytotoxicity assays, such as WST-1 viability tests [8,13]. It remains controversial to what extent these effects originate from the intrinsic properties of the hydrogel network, from residual or leachable components released during swelling, from processing-related parameters of the silicone matrix, or from interactions between both materials in the hybrid system. Diverging hypotheses exist in the literature, particularly regarding the relevance of extractables and leachables for cytotoxic responses and long-term material stability [8,14,15,16], yet comprehensive studies that jointly address swelling behavior, processing effects, and in vitro cytotoxicity within a unified experimental framework remain scarce.

To overcome these performance and biocompatibility limitations, both the hydrogel chemistry and the manufacturing process must be systematically optimized. Previous hydrogel iterations based on predominantly acidic, non-neutralized formulations showed limited swelling performance and raised concerns regarding the use of a highly acidic material in contact with biological environments. To address this, a modified synthesis strategy was implemented in which the monomer solution was partially neutralized with sodium hydroxide to near-neutral pH prior to polymerization. The resulting incorporation of mobile counterions promotes osmotic water uptake and swelling while avoiding the strongly acidic conditions of the previous formulation. This modified hydrogel matrix was therefore selected as the baseline material for integration into the elastomeric silicone network. Furthermore, processing parameters such as silicone curing conditions may affect crosslink density, residual catalysts, or trapped air inclusions, while post-treatment steps of the hydrogel, especially washing procedures, can significantly alter swelling kinetics and the presence of leachable species [17,18]. To realize such tailored hybrid structures, extrusion-based additive manufacturing provides a suitable processing route, as it enables spatially defined deposition of the composite and may ultimately facilitate its local integration onto silicone-based electrode carriers.

Consequently, a systematic qualification of hydrogel–silicone bimorphs is required in which material-related and process-induced influences are deliberately varied, quantified, and clearly separated. The aim of this work is therefore the experimental qualification and optimization of a swellable hydrogel–silicone bimorph with respect to reproducible swelling behavior, material stability, and cell viability. Particular emphasis is placed on the influence of silicone curing regimes and hydrogel post-treatment by solvent washing. To disentangle material- and process-related effects, silicone reference samples without hydrogel are investigated alongside the hybrid system. Swelling characteristics, mass and volume changes, and cell-based viability are systematically analyzed, with a focus on identifying the role of potential leachables.

## 2. Materials and Methods

Materials, Reagents & Consumables

- Hydrogel powder (VPA-AAm): Synthesized VPA-AAm copolymer;
- Absolute ethanol (99.8%);
- Medical-grade liquid silicone rubber, NuSil MED-4850, NuSil Technology, Carpinteria, CA, USA;
- Rhodamine B (C.I. 45170), PanReac AppliChem ITW Reagents, Darmstadt, Germany;
- Ringer’s Infusion solution B, Braun SE, Melsungen, Germany;
- Dulbecco’s Modified Eagle Medium (DMEM), Bio & Sell, Feucht, Germany;
- Fetal bovine serum (FBS), F7524, Sigma-Aldrich, St. Louis, MO, USA;
- NIH-3T3 mouse fibroblasts, German Collection of Microorganisms and Cell Cultures (DSMZ), Braunschweig, Germany;
- Trypsin/Ethylenediaminetetraacetic acid (EDTA), Bio & Sell, Feucht, Germany;
- Dimethyl sulfoxide (DMSO), PanReac AppliChem ITW Reagents, Darmstadt, Germany;
- WST-1 reagent, Roche Applied Science, Mannheim, Germany.

Equipment & Software

- Planetary ball mill: Pulverisette 23, Fritsch, Idar-Oberstein, Germany;
- Vibratory sieve shaker: Analysette 3 Pro, Fritsch, Idar-Oberstein, Germany;
- Centrifugal mixer: Dual asymmetric centrifugal mixer (Speedmixer DAC-150), Hauschild, Hamm, Germany;
- CAD software (Version 24.3, Build U.61.0.0): Onshape, Parametric Technology Corporation Inc., Boston, MA, USA;
- 3D printer: 3D bioplotter, EnvisionTEC, Gladbeck, Germany;
- Vacuum oven: Vacutherm VT 6025, Heraeus, Hanau, Germany;
- Autoclave: SHP Steritec AG, Dettighofen, Switzerland;
- Drying oven: UF 750, Memmert, Schwabach, Germany;
- Rotary shaker: KS 130 Basic, IKA, Staufen, Germany;
- Cryogenic mill: CryoMill, Retsch, Haan, Germany;
- Fluorescence microscope: BZ-X810, Keyence, Osaka, Japan;;
- Drying oven: BE 400, Memmert, Schwabach, Germany;
- Confocal Raman microscope: WITec alpha300RA (532 nm laser), WITec GmbH, Ulm, Germany;
- Humidified CO_2_ incubator: Binder, Tuttlingen, Germany;
- Microplate reader: Biotek Synergy H1, Agilent, Winooski, VT, USA;
- GraphPad Prism Version 10.6.1, GraphPad Software, Boston, MA, USA.

### 2.1. Material Preparation and Processing

#### 2.1.1. Hydrogel Synthesis

To fabricate functional hydrogel–silicone bimorph actuators, the embedded hydrogel must exhibit highly predictable, ion-driven swelling behavior alongside structural stability. Commercially available hydrogels, such as non-ionic or partially pre-formulated acrylate/acrylamide-based networks, often present severe limitations for target biomedical integrations. These standard formulations frequently contain either free, highly acidic monomer groups or unoptimized counterion profiles. In a purely acidic state, the lack of alkali metal cations significantly restricts the osmotic pressure inside the polymer network, leading to insufficient and unpredictable swelling capacity due to the absence of well-defined hydration shells. Furthermore, embedding highly acidic components directly into medical-grade silicone matrices is fundamentally counterproductive for biomedical applications, where tissue contact demands physiological compatibility.

A tailored vinylphosphonic acid–acrylamide (VPA–AAm) hydrogel was synthesized using VPA and AAm at a molar ratio of 1:1 and N,N′-methylenebisacrylamide (MBAM) as crosslinker (2.0 mol% relative to the monomers). Prior to polymerization, the VPA solution was partially neutralized with sodium hydroxide (NaOH) to pH 6.8. Polymerization was initiated using tetramethylethylenediamine (TMEDA) and potassium peroxodisulfate (KPS) following deoxygenation under argon and proceeded for 22 h at ambient temperature. The resulting hydrogel was subsequently purified by repeated solvent exchange with isopropanol, dried under reduced pressure, and mechanically ground to obtain a powder. The VPA–AAm hydrogel was synthesized by Unavera ChemLab GmbH (Mittenwald, Germany). A complete synthesis protocol, including reagent quantities, concentrations, addition sequence, reaction conditions, and purification procedure, is provided in the Supplementary Materials (Section S1).

#### 2.1.2. Sample Preparation

A precisely weighed amount (10 g) of hydrogel powder (VPA-AAm) was suspended in a 1:10 ratio of absolute ethanol (99.8%) (100 mL), allowing extraction for a defined period (1 h) under static conditions. After sedimentation of the solid phase, the supernatant containing dissolved low-molecular-weight substances was decanted. The remaining solid residue was dried under vacuum (40 °C, 250 mbar) for 24 h.

For material preparation, the hydrogel powder was mechanically processed in separate batches for its washed and unwashed states, respectively. Comminution was performed using a planetary ball mill (Pulverisette 23, Fritsch, Idar-Oberstein, Germany) at a frequency of 30 s^−1^, divided into a 30 min coarse grinding step followed by an additional 30 min fine grinding cycle. The milled powder was subsequently fractionated using a vibratory sieve shaker (Analysette 3 Pro, Fritsch, Idar-Oberstein, Germany) into particle size fractions of >100 µm, 50–100 µm, and 20–50 µm to obtain a defined and uniform particle size distribution.

Medical-grade liquid silicone rubber (NuSil MED-4850, NuSil Technology, Carpinteria, CA, USA) was mixed with the dried and sieved hydrogel fraction (20–50 µm) using a dual asymmetric centrifugal mixer (Speedmixer DAC-150, Hauschild, Hamm, Germany). Composite formulations were prepared with hydrogel contents of 25, 30, and 35 wt.%. In addition, pure silicone samples were produced as reference specimens.

Rectangular samples (55 × 13 mm, thickness 1 mm) were designed using CAD software (Onshape, Parametric Technology Corporation Inc., Boston, MA, USA) and fabricated with a 3D bioplotter (EnvisionTEC, Hamm, Germany). Following 3D printing, the specimens were vacuum-degassed at 250 mbar for 3–6 h prior to thermal curing in a vacuum oven (Vacutherm VT 6025, Heraeus, Hanau, Germany) at 150 °C for 5 min. Additionally, for the swelling tests, square specimens (13 × 13 mm, thickness 1 mm) were fabricated in triplicate (n = 3) for each hydrogel concentration using both washed and unwashed hydrogel fractions.

### 2.2. Sterilisation

Following the 3D-printing and thermal curing procedures, specimens intended for the subsequent in vitro cytotoxicity assessment were sterilized by autoclaving (SHP Steritec AG, Switzerland) at 121 °C for 20 min. Subsequently, these samples were dried in a drying oven (UF 750, Memmert, Schwabach, Germany) at 45 °C and stored until further use.

Specimens used for the swelling experiments were not autoclaved. The composites had previously been thermally cured at 150 °C according to the silicone processing conditions, i.e., above the temperature applied during autoclaving. Furthermore, the specimens were individually enclosed in sterilization pouches during autoclaving, and no macroscopically visible swelling was observed after the procedure. Although subtle effects of the humid and pressurized autoclave environment on the hydrogel–silicone composite cannot be excluded, no apparent structural alteration was observed.

No dedicated chemical or thermal characterization was performed before and after autoclaving; therefore, subtle sterilization-induced changes that were not macroscopically apparent cannot be excluded.

### 2.3. Preparation of Fluorescently Labeled Hydrogel Particles

To visualize the hydrogel network and evaluate its distribution within the silicone matrix, the hydrogel was intrinsically labeled prior to composite formulation. A Rhodamine B solution (100 µg/mL) was prepared by dissolving 2 mg of the dye (C.I. 45170, PanReac AppliChem ITW Reagents, Darmstadt, Germany) in 20 mL of deionized water. Subsequently, 2 g of raw hydrogel powder was immersed in this solution and incubated on a rotary shaker (KS 130 Basic, IKA, Staufen, Germany) for 24 h at room temperature, protected from light. Following the loading process, the hydrogel was dried to a constant mass in a vacuum oven at 40 °C for six days. The fully dehydrated, dyed hydrogel was then pulverized using a cryogenic mill (CryoMill, Retsch, Haan, Germany) and sieved to isolate the target fraction (20–50 µm). These labeled particles were subsequently incorporated into the NuSil silicone matrix at a concentration of 30 wt.% and processed via the established 3D-printing method.

### 2.4. Fluorescence Microscopy

The phase morphology and particle distribution of the printed composites were evaluated using a fluorescence microscope (BZ-X810, Keyence, Osaka, Japan) under green light excitation. A heat-cured specimen (cured at 150 °C for 5 min) was analyzed via cross-section prepared with a scalpel. Complementary chemical imaging of the composite morphology and hydrogel distribution was performed by Raman spectroscopy, as described in Section 2.6.

This qualitative analysis focused on identifying differences in particle arrangement, potential agglomeration, or the formation of a percolating network induced by the respective processing state.

### 2.5. Swelling Test

To evaluate the swelling behavior of the printed composites, the specimens were immersed in Ringer’s solution (Braun SE, Melsungen, Germany) for a total duration of 28 days. Ringer’s solution was used to better replicate the physiological environment. For each test, the initially weighed dry samples were placed into individual 10 mL polypropylene conical Falcon tubes filled with 10 mL of Ringer’s solution. Throughout the entire experiment, the containers were stored in a drying oven (BE 400, Memmert, Schwalbach, Germany) maintained at a constant temperature of 37 °C to simulate physiological conditions.

During the initial phase of immersion, the sample weights were recorded at 1-h intervals for the first eight hours. Prior to each gravimetric measurement, the specimens were carefully removed from the solution and gently blotted with a lint-free laboratory wipe to eliminate any excess surface liquid. Following the initial phase of hourly measurements, mass tracking was performed every 24 h for seven consecutive days, followed by weekly measurements until the final 28-day mark. To maintain consistent osmotic conditions and prevent saturation effects, the Ringer’s solution was renewed after 2 weeks and 3 weeks of total immersion time.

The weight of each specimen was systematically recorded in both its initial dry state (*W*_0_) and its respective swollen states (*W_t_*) to determine the swelling rate (S) according to Equation (1):

(1) $$S\;=\;\frac{W_{t}-W_{0}}{W_{0}}\times 100$$

Note that S fundamentally represents a mass-based swelling ratio. Due to the near-identical densities of the hydrogel and PDMS phases (*ρ* ≈ 1.0 g/cm^3^), this value serves as a direct approximation of the volumetric swelling ratio.

### 2.6. Raman Spectroscopy

Confocal Raman spectroscopy was employed to analyze the phase distribution, interfacial morphology, and potential leaching behavior of the hydrogel–silicone composites. All spectra were recorded using a WITec alpha300RA Raman microscope (WITec GmbH, Ulm, Germany) equipped with a 532 nm excitation laser.

For spatial imaging of the composite microstructures, two-dimensional area scans were performed over a region of 100 × 100 μm^2^ with a spatial resolution of 100 × 100 pixels. Spectra were acquired with an integration time of 0.1 s per pixel at a laser power of 25 mW. Hyperspectral dataset post-processing included cosmic ray removal and intensity normalization to the characteristic C − H stretching band of the polydimethylsiloxane (PDMS) matrix at 2908 cm^−1^ (represented in greyscale). Amide-containing hydrogel domains (represented in red) were selectively mapped by tracking the intensity of the amide peak at 1617 cm^−1^.

To evaluate potential leaching into the swelling medium, composite specimens were swollen in 15 mL of deionized water for approximately 70 h. Raman spectroscopic measurements were performed on the immersion liquids at a laser power of 55 mW, using an integration time of 10 s with 10 accumulations. Spectral profiles of the swelling water residues were directly evaluated against an identically processed pure deionized water blank. For the loading-dependent comparison of released species, the relative amplitude of the band at approximately 1408 cm^−1^ was evaluated following H_2_O-blank subtraction and OH-normalization.

### 2.7. In Vitro Cytotoxicity Assessment

To evaluate the potential cytotoxicity of the fabricated hydrogel/silicone composites, an in vitro cell viability analysis was conducted utilizing the water-soluble tetrazolium salt (WST-1) assay, which photometrically measures the metabolic activity of viable cells.

Due to the high water absorption capacity of the hydrogel phase, cytotoxicity testing was performed using extract media instead of direct contact testing. Extract preparation was carried out guided by DIN EN ISO 10993-12 [19]. The volume of extraction medium was calculated in accordance with Table 1 of DIN EN ISO 10993-12 [19] based on the sample surface area, maintaining a surface area-to-volume ratio of 3 cm^2^/mL (thickness 0.5 to 1.0 mm). Specifically, one sterilized specimen with a total surface area of 15 cm^2^ was placed into a 15 mL tube and incubated with 5 mL of phenol red-free Dulbecco’s Modified Eagle Medium (DMEM, Bio & Sell, Feucht, Germany) supplemented with 10% fetal bovine serum (FBS, F7524, Sigma-Aldrich, St. Louis, MO, USA). A blank extract containing 5 mL of the supplemented DMEM was incubated under identical conditions. To ensure the near-complete extraction, an extended incubation period of 120 h at 37 °C and 5% CO_2_ in a humidified incubator (Binder, Tuttlingen, Germany) was selected to provide an additional margin for the extraction of potentially leachable species. NIH-3T3 mouse fibroblasts (German Collection of Microorganisms and Cell Cultures, Braunschweig, Germany) were cultured in DMEM supplemented with 10% FBS at 37 °C and 5% CO_2_ under standard sterile conditions. Cells were passaged every 2–3 days using trypsin/ethylenediaminetetraacetic acid (EDTA) solution (Bio & Sell, Feucht, Germany).

For the WST-1 assay, 10,000 fibroblasts per well were seeded into a 96-well plate in a volume of 100 µL of standard culture medium and incubated for 24 h at 37 °C and 5% CO_2_ to allow for cell adhesion. Thereafter, the medium was replaced with 100 µL of the respective conditioned extract media, and the cells were incubated for 24 h. For each material composition, a single extract was prepared, from which five biological replicates (independent wells) were tested. Statistical differences in cell viability among the investigated extracts were evaluated by one-way ANOVA followed by Dunnett’s multiple-comparisons test using the blank extract as the reference. Statistical significance was set at *p* ≤ 0.05. Cells cultured in DMEM without phenol red supplemented with 10% FBS served as the blank extract, while cells treated with 10% dimethyl sulfoxide (DMSO; PanReac AppliChem ITW Reagents, Darmstadt, Germany) in medium were used as the positive control.

Subsequently, 10 µL of WST-1 reagent (Roche Applied Science, Mannheim, Germany) was added to each well, followed by incubation for 60 min at 37 °C and 5% CO_2_. After incubation, the cleavage of the tetrazolium salt WST-1 to formazan was quantified using a microplate reader (Biotek Synergy H1, Agilent, Santa Clara, CA, USA) at a measurement wavelength of 450 nm against a reference wavelength of 650 nm. The measured absorbance directly correlates with the number of metabolically active, viable cells. Cell viability was calculated from the mean optical density of each sample relative to the blank extract, which was defined as 100% viability.

## 3. Results

### 3.1. Sample Preparation

#### 3.1.1. Hydrogel Processing

During the preparation of the VPA-AAm powder, achieving a homogeneous particle size distribution proved to be challenging. The objective was to obtain a defined particle fraction in the range of 20–50 µm to ensure a uniform distribution within the silicone matrix and guarantee reproducible material properties. Since the dried hydrogel initially exhibited a broad particle size distribution after the primary milling process, the grinding procedure had to be repeated multiple times.

Notably, the pre-washed powder was significantly easier to sieve following the drying process. This treatment led to a higher yield of the target 20–50 µm fraction, as it reduced particle agglomeration and enhanced the efficiency of the sieving steps, ultimately providing a sufficient quantity of fine material suitable for the subsequent 3D-printing process.

#### 3.1.2. Morphological Analysis and Visual Appearance of 3D-Printed Samples

**Figure 1:**
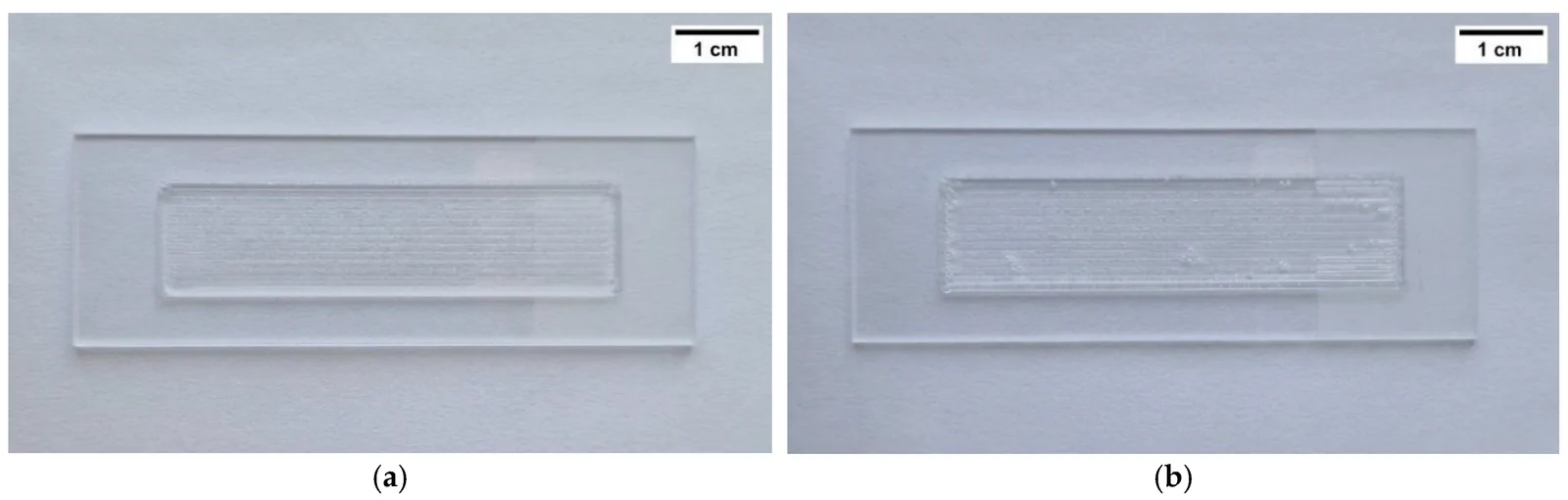
Visual appearance of 3D-printed pure silicone samples under different conditions: (**a**) uncured; (**b**) heat-cured in a vacuum oven (150 °C, 5 min).

**Figure 2:**
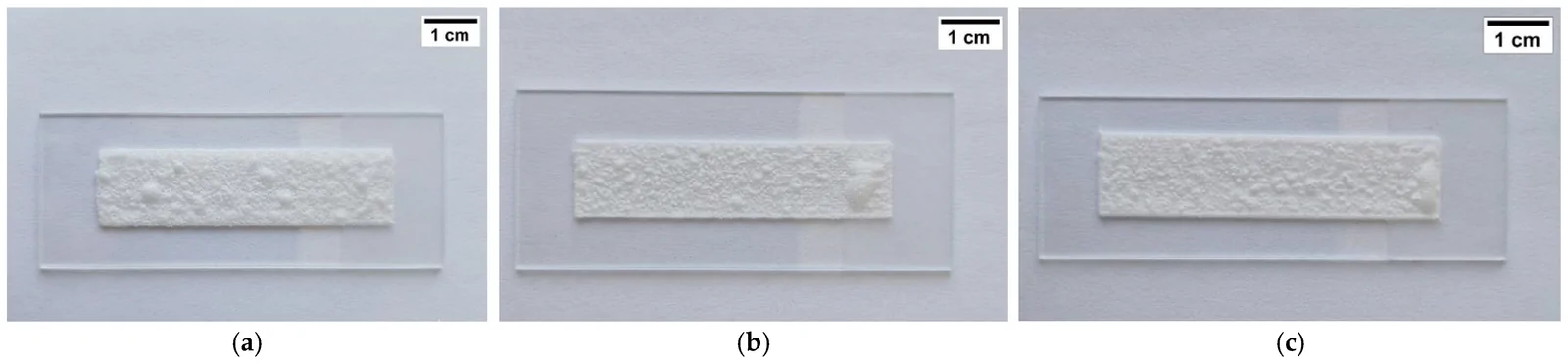
Visual appearance of 3D-printed hydrogel/silicone composites, heat-cured in a vacuum oven (150 °C, 5 min), formulated with VPA-AAm hydrogel at filler loadings of (**a**) 25 wt.%, (**b**) 30 wt.%, and (**c**) 35 wt.%.

**Figure 3:**
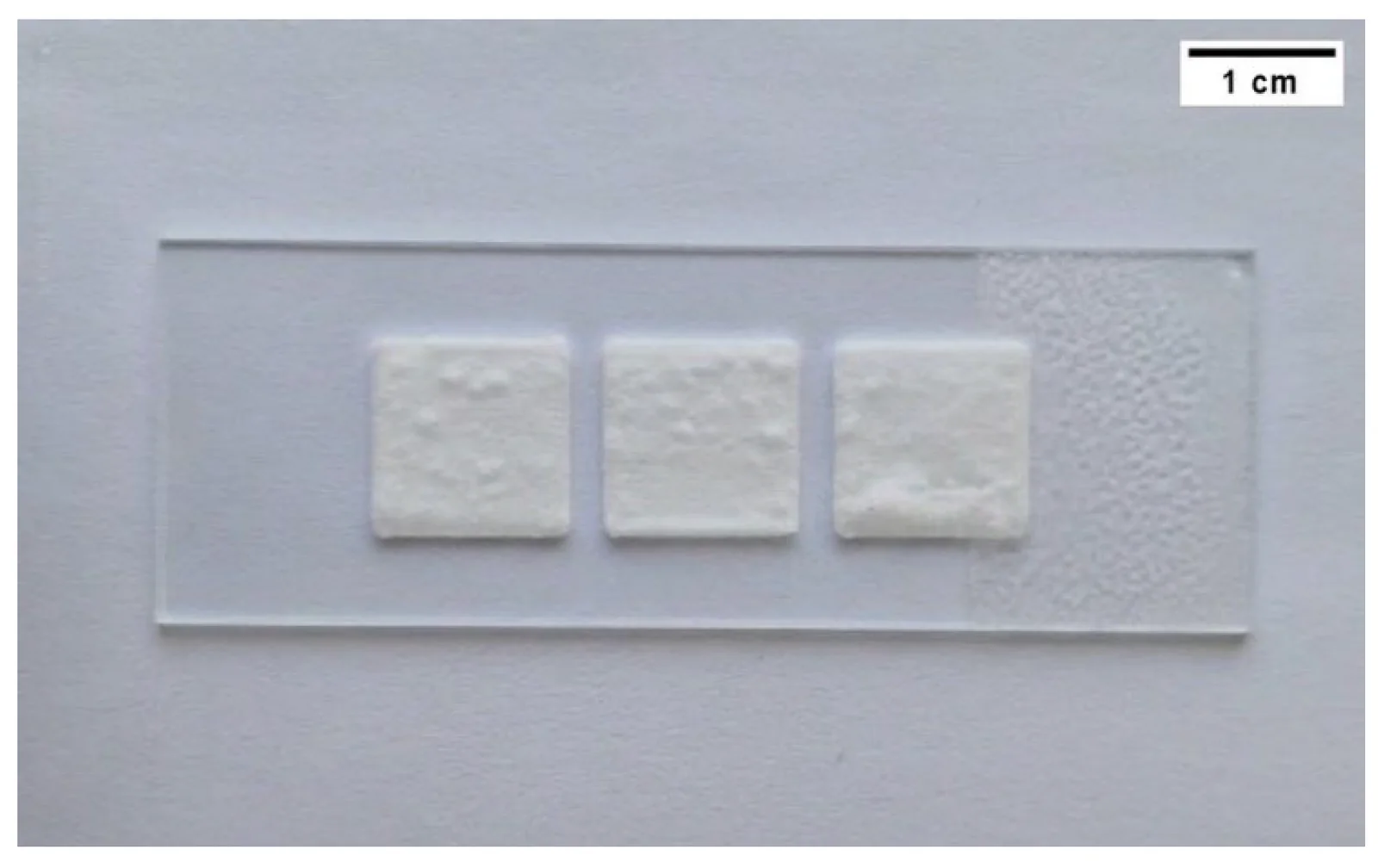
Visual appearance of 3D-printed hydrogel/silicone composite samples fabricated for the swelling test, heat-cured in a vacuum oven (150 °C, 5 min).

The visual characteristics of the 3D-printed samples are illustrated in Figure 1, Figure 2 and Figure 3. The neat uncured NuSil silicone specimens (Figure 1) (a) exhibited a high degree of transparency and a smooth, defect-free surface. In contrast, the neat specimens cured at 150 °C for 5 min (b) displayed a low density of small, isolated bubbles. Notably, even though all mixtures were subjected to a vacuum degassing step prior to curing, the formation of gas inclusions was significantly more pronounced in all hydrogel composites (25, 30, and 35 wt.%) when exposed to the 150 °C oven-curing process (Figure 2 and Figure 3).

The microscopic analysis revealed that the composite matrix contained internal voids or bubbles. This porosity was not fully eliminated by the preceding vacuum degassing step. Furthermore, a slight increase in bubble density was observed with higher hydrogel concentrations.

Regarding the optical properties, all composite specimens reached complete opacity at the initial loading of 25 wt.% VPA-AAm. No visually detectable difference in transparency was observed between the 25, 30, and 35 wt.% samples, which all exhibited a uniform milky-white appearance. This interpretation suggests that the threshold for total light scattering, caused by the refractive index mismatch between the silicone and the hydrogel, is already exceeded at a loading of 25 wt.%. Consequently, for these composite systems, the filler concentration can be adjusted for mechanical or chemical properties without further altering the macroscopic optical appearance of the final part.

### 3.2. Phase Morphology and Hydrogel Distribution

The fluorescent labeling of the hydrogel powder utilizing Rhodamin B proved highly successful, yielding an intense fluorescence signal that was clearly visible during the microscopic investigation. This effective staining provided a sharp contrast against the non-fluorescent silicone matrix, allowing for a precise evaluation of the internal structures.

**Figure 4:**
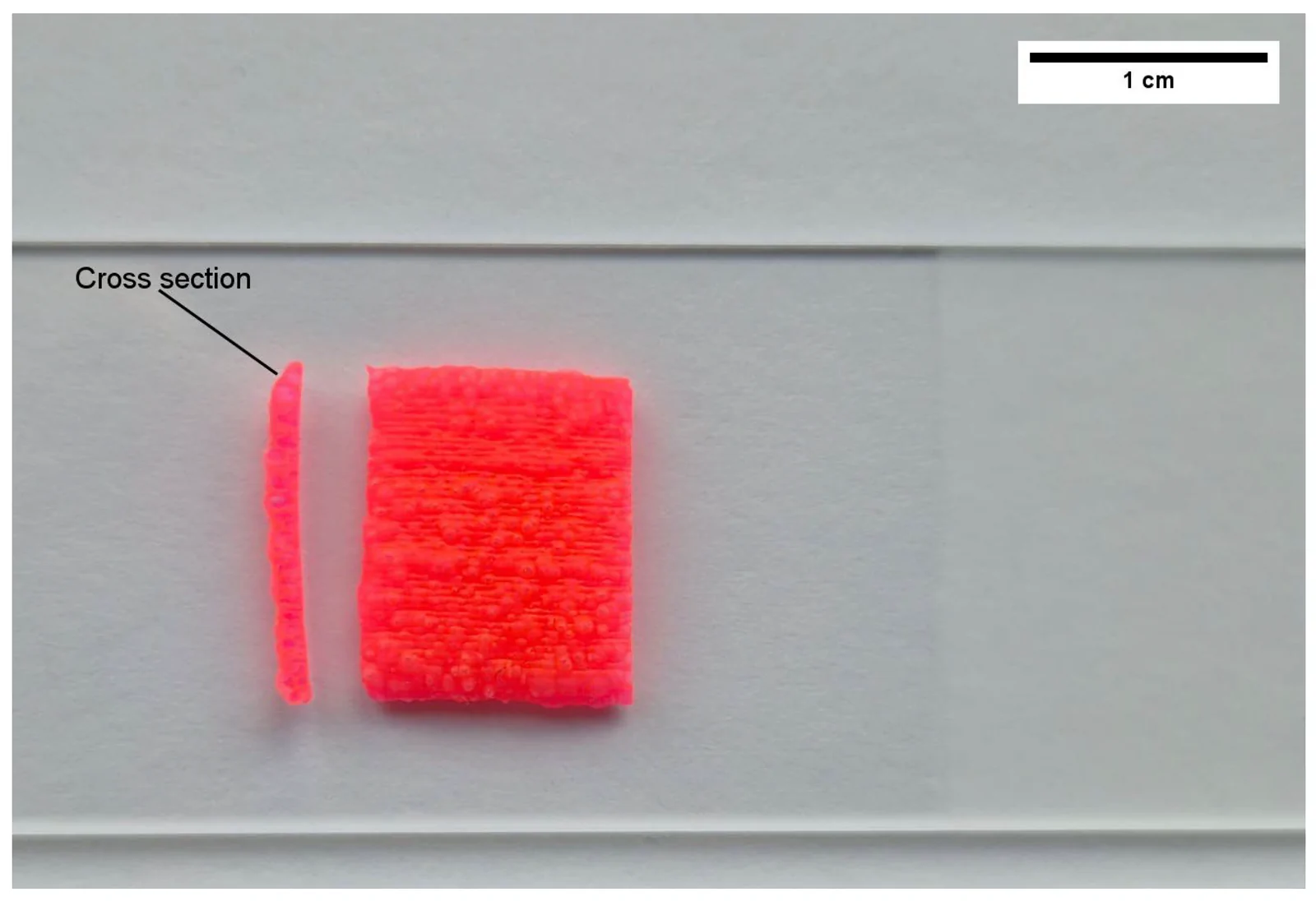
3D-printed silicone composite sample containing 30 wt.% Rhodamine B-stained VPA-AAm hydrogel particles. The separated slice on the left indicates the cross-section prepared for subsequent fluorescence microscopic analysis of the internal composite morphology.

**Figure 5:**
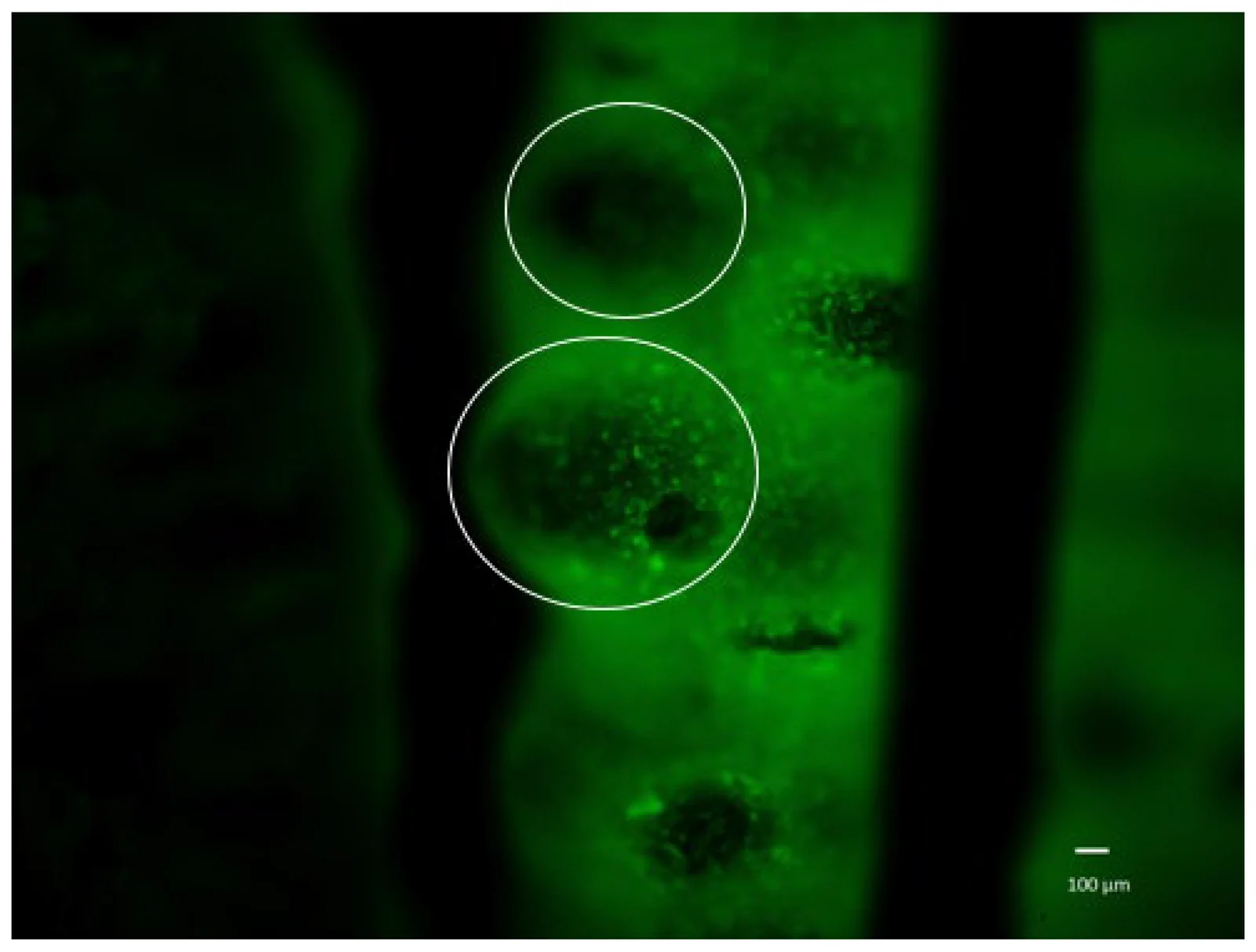
Fluorescence micrograph of a cross-sectioned, 3D-printed, heat-cured silicone composite sample containing 30 wt.% Rhodamine B-stained VPA-AAm hydrogel particles. Cross-sectioning exposed microscopic voids formed during thermal curing, two representative examples of which are highlighted by circles. The opened voids provide a view of their internal surfaces, revealing individual fluorescent hydrogel particles embedded and dispersed within the surrounding PDMS matrix.

Macroscopic evaluation of the prepared composite sample (Figure 4) and subsequent fluorescence microscopic imaging of the cross-sectioned state (Figure 5) revealed a highly homogeneous distribution of the hydrogel phase throughout the composite. Notably, regarding the spatial arrangement, the morphology did not exhibit any signs of a continuous, interconnected or percolating network structure. Instead, the hydrogel phase was strictly present as well-dispersed, individual particles that remained isolated from one another within the polymeric matrix, showing no significant agglomeration. In terms of particle dimensions, the size of these individual domains consistently remained within the expected range of 20–50 µm. This confirms that the original particle size distribution, which was predefined by the mesh size during the initial mechanical sieving process, was successfully maintained during both the printing and the subsequent curing procedures.

**Figure 6:**
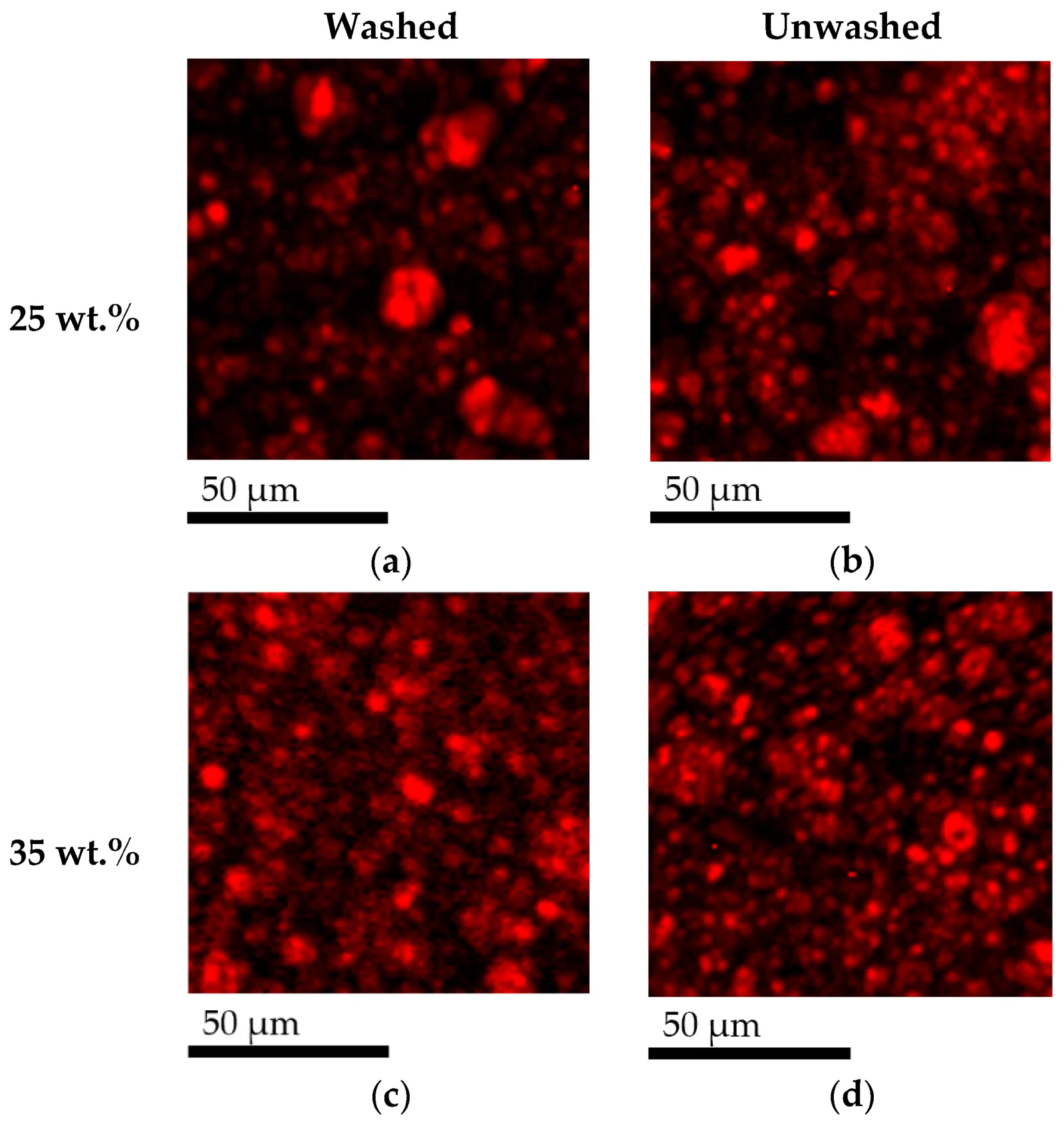
Raman imaging of amide-rich regions using the amide-associated band at 1617 cm^−1^ to distinguish the continuous silicone matrix from spatially confined hydrogel-rich domains. Maps are shown for the available 25 wt.% hydrogel formulation in washed (**a**)/unwashed (**b**) processing conditions and 35 wt.% hydrogel formulation in washed (**c**)/unwashed (**d**) processing conditions.

Complementing the optical analysis, Raman imaging provided a chemically selective confirmation of the composite microstructure (Figure 6). Mapping of the PDMS-associated band at 2908 cm^−1^ indicated a continuous silicone matrix, whereas the amide-associated band at 1617 cm^−1^ identified spatially confined hydrogel-rich domains embedded within the matrix. The dimensions of the Raman-identified domains were consistent with the nominal 20–50 µm hydrogel particle fraction used for composite preparation. At higher hydrogel loading, the spatial density of hydrogel-associated domains increased, resulting in reduced characteristic distances between neighboring domains within the continuous PDMS phase.

### 3.3. Swelling Behavior and Leaching Analysis

**Figure 7:**
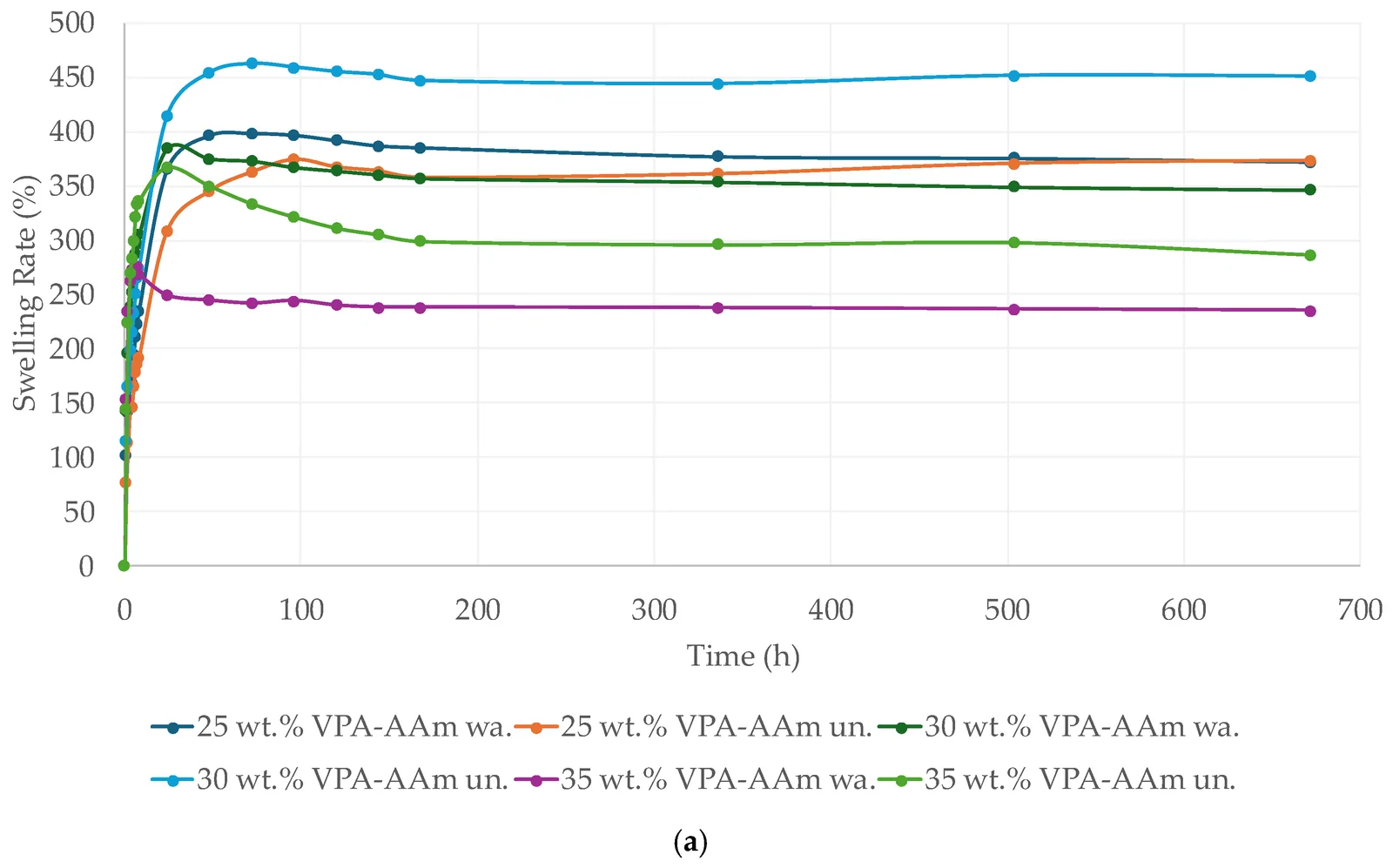

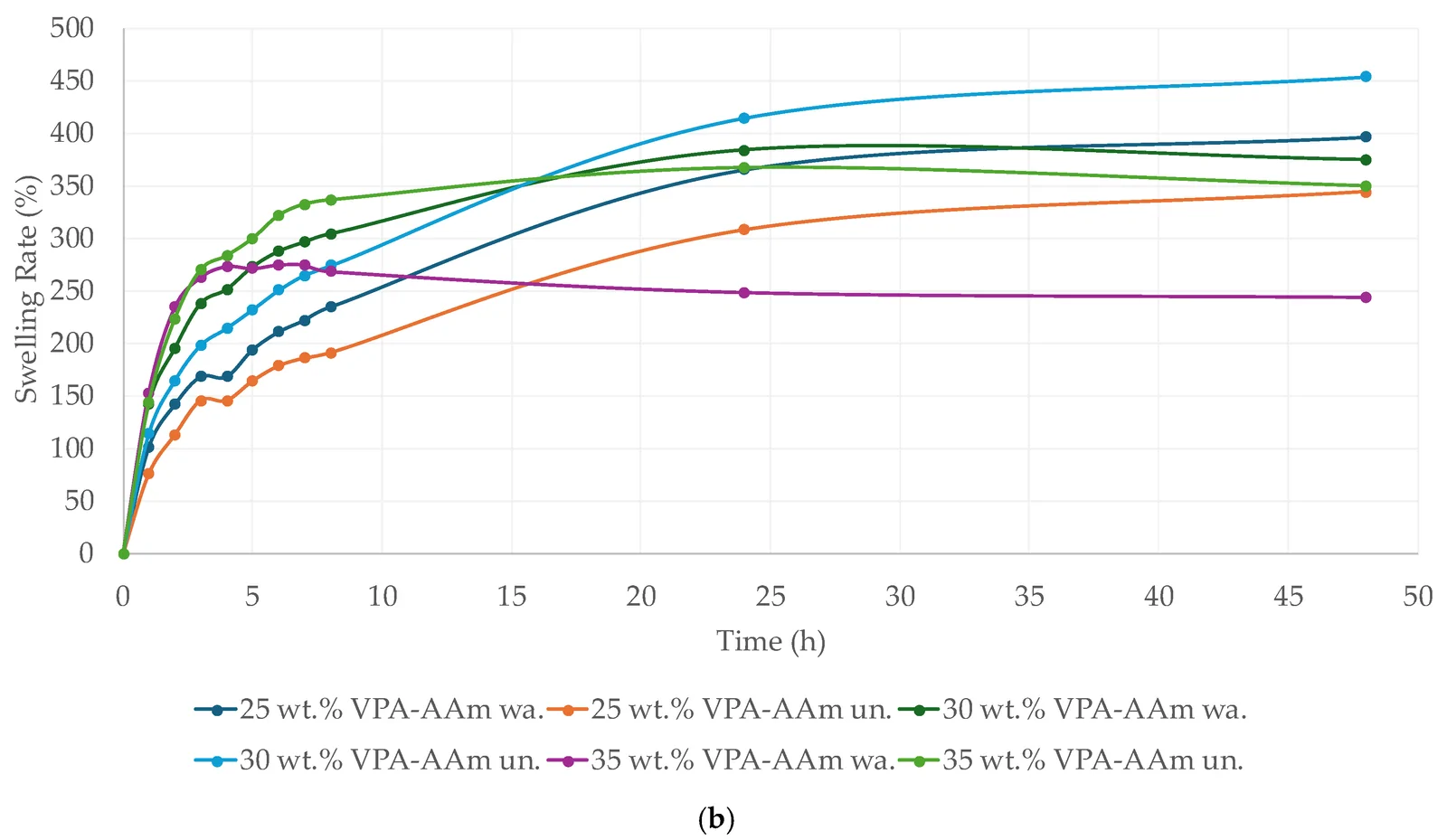
Swelling rate of the 3D-printed composite formulations as a function of time: (**a**) long-term swelling kinetics up to 28 days (672 h) and (**b**) detailed initial swelling behavior during the first 48 h. The thermally cured (150 °C, 5 min) silicone composites were filled with 25, 30, and 35 wt.% of washed (wa.) or unwashed (un.) VPA-AAm hydrogel particles.

The gravimetric swelling kinetics and the maximum swelling capacity of the 3D-printed hydrogel/silicone composites were evaluated over a total duration of 4 weeks (28 days). The resulting swelling profiles, recorded at hourly (1–8 h), daily (1–7 days), and weekly (2–4 weeks) intervals, are presented in Figure 7, which consists of two separate diagrams: one illustrating the complete 28-day swelling profile (a) and a detailed close-up focusing specifically on the first 48 h (b). All investigated composite formulations exhibited a characteristic biphasic swelling kinetic. The first phase, spanning from 1 to 8 h, was characterized by a rapid and steep increase in the swelling rate across all specimens. Between 24 and 48 h (days 1–2), the water uptake transitioned into a stable plateau phase, which was subsequently maintained without significant fluctuations until the end of the 4-week testing period.

Regarding the mass fraction of the embedded hydrogel particles, a non-linear effect on the equilibrium swelling rate was observed. The 30 wt.% hydrogel loading achieved the highest overall water absorption within the study, where the unwashed formulation reached the absolute maximum swelling rate of approximately 450–460% at days 2–3, remaining stable thereafter, while the washed counterpart stabilized at a constant plateau between 370% and 400% from day 1 onwards. In contrast, specimens containing the lowest filler content of 25 wt.% displayed a steady increase during the first 8 h and reached a stable equilibrium at approximately 375% after one day. Beyond this point, the curves for both the washed and unwashed states of the 25 wt.% formulation converged and remained completely constant up to week 4. A further increase in the filler content to 35 wt.%, however, resulted in a distinct decrease in swelling performance. The washed 35 wt.% specimens reached a premature plateau after only 3 h, stabilizing at a significantly lower swelling rate of 240–275%, whereas the unwashed 35 wt.% specimens recorded a temporary maximum of approximately 330% at 7 h, followed by a continuous, gradual decline over the subsequent days and weeks, finalising at approximately 290% after 4 weeks.

The purification state of the hydrogel phase furthermore exerted a concentration-dependent effect on the swelling profiles. For both the 30 wt.% and 35 wt.% formulations, the unwashed specimens consistently exhibited a higher swelling capacity than the corresponding washed samples throughout the measurement period. For the 35 wt.% unwashed sample specifically, a characteristic long-term decay in the swelling rate was prominent following its peak at 7 h. Conversely, for the 25 wt.% formulation, the influence of the washing step became entirely negligible after the first 24 h, as both curves converged completely into the identical equilibrium plateau.

**Figure 8:**
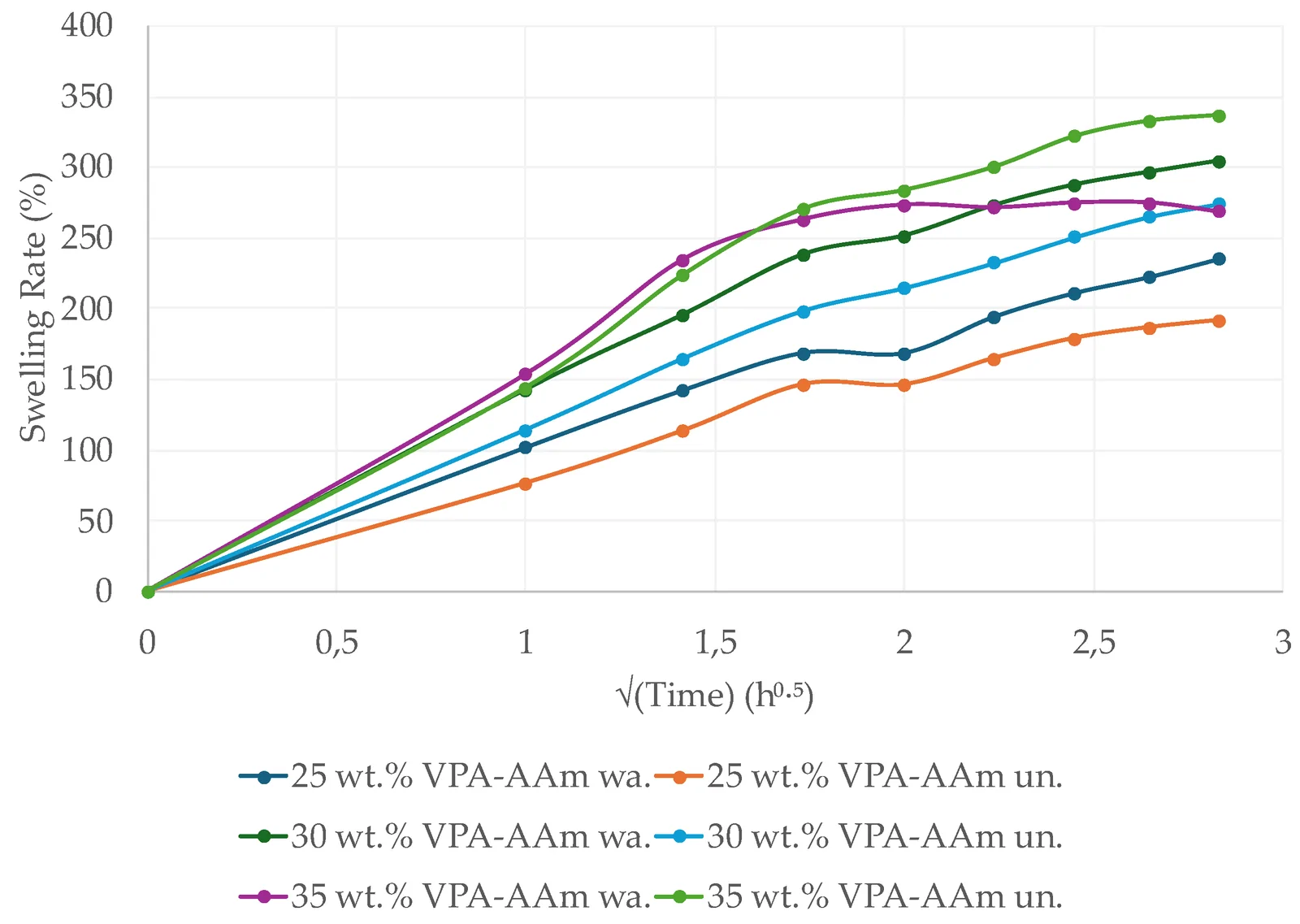
Swelling rates of the 3D-printed composite formulations plotted against the square root of time ($\sqrt{t}$). The thermally cured (150 °C, 5 min) silicone composites were filled with 25, 30, and 35 wt.% of washed (wa.) or unwashed (un.) VPA-AAm hydrogel particles.

To investigate the underlying mass transport kinetics, the swelling ratio was plotted against the square root of time (h^0.5^) as shown in Figure 8. All formulation curves display a non-linear trajectory in the initial stages before transitioning into a steady plateau phase. The velocity of the swelling process scales with the filler concentration, with the 35 wt.% formulations demonstrating the steepest initial slopes.

To determine accurate apparent diffusion coefficients (*D_app_*), the quantitative kinetic analysis was restricted to the initial short-term approximation range where *M_t_*/*M_∞_* ≤ 0.6.

The resulting values, summarized in Table 2, reveal distinct quantitative trends regarding the mass transport velocity. Within the washed sample series, *D_app_* scales progressively with increasing filler loading, accelerating from 2.02 × 10^−5^ cm^2^ ⋅ h^−1^ at 25 wt.% to a maximum of 1.53 × 10^−4^ cm^2^ ⋅ h^−1^ at 35 wt.%. Furthermore, the purification state exerts a systematic effect across all formulations: washed networks consistently exhibit higher diffusion coefficients than their respective unwashed counterparts. This acceleration is most pronounced in the intermediate 30 wt.% formulation, where the apparent diffusion coefficient increases by a factor of three upon washing (from 2.15 × 10^−5^ to 6.44 × 10^−5^ cm^2^ ⋅ h^−1^).

**Figure 9:**
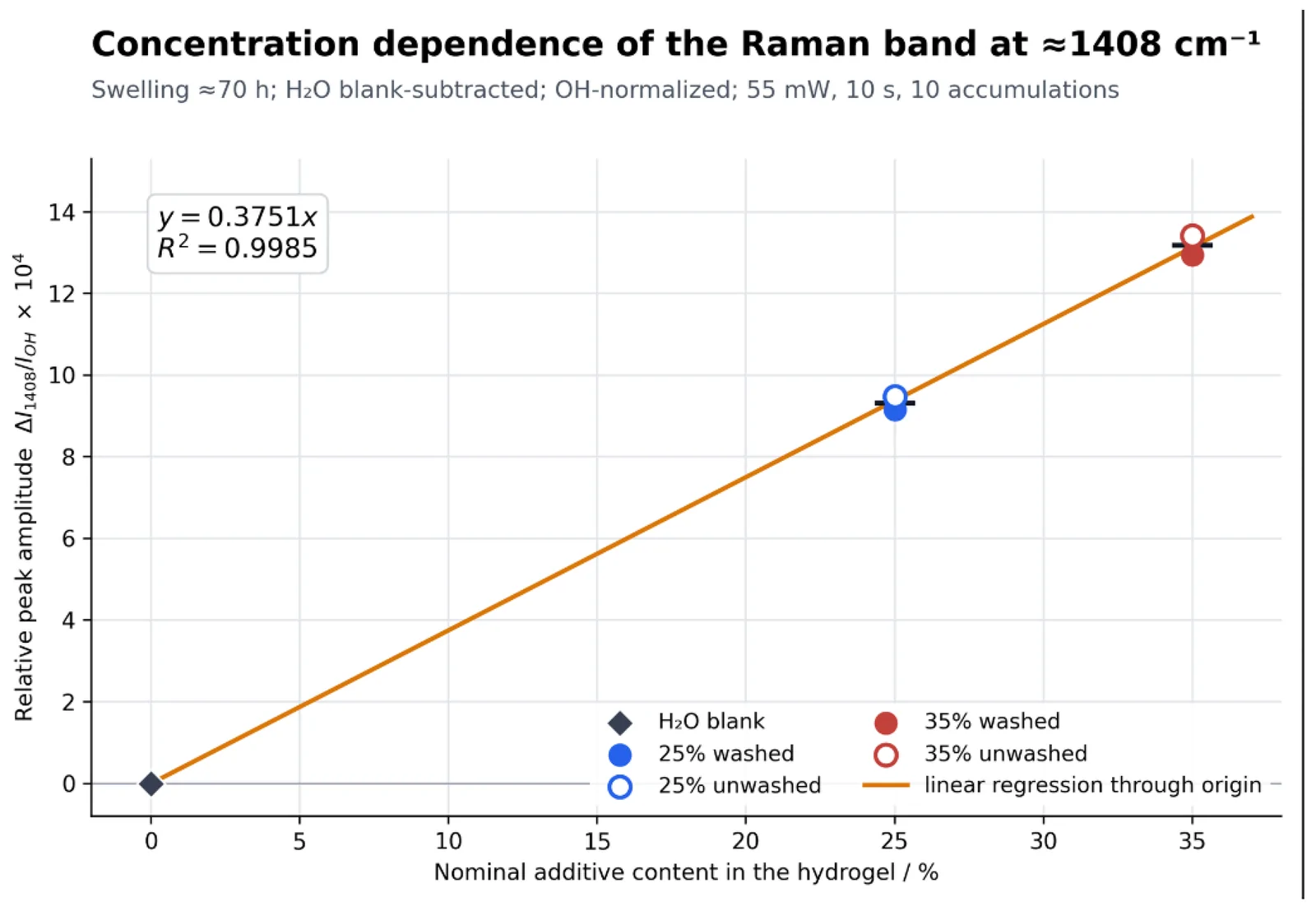
Relative Raman-band amplitude at approximately 1408 cm^−1^ in the swelling medium after approximately 70 h, following H_2_O-blank subtraction and OH normalization. The signal increased with nominal hydrogel content, whereas washed and unwashed samples at the same loading showed only minor differences. The regression shown is constrained through the origin and is intended as a descriptive representation of the loading-dependent signal.

Complementing the gravimetric analysis, Raman analysis of the swelling medium after approximately 70 h confirmed the release of hydrogel-associated species from the composites (Figure 9). The band observed at approximately 1408 cm^−1^ was assigned to the symmetric stretching vibration of carboxylate groups, νs(COO^−^), associated with released VPA–AAm hydrogel-derived species [20]. In the corresponding hydrogel reference spectrum, this band was observed at approximately 1415 cm^−1^. The shift toward lower wavenumbers in the aqueous phase is consistent with changes in the local chemical environment upon dissolution, particularly hydration and solvation as well as altered ion–carboxylate interactions [21]. The relative amplitude of the band at approximately 1408 cm^−1^ increased with nominal hydrogel content, with the 35 wt.% samples exhibiting a higher signal than the 25 wt.% samples. At a given hydrogel loading, only minor differences were observed between washed and unwashed samples.

These measurements demonstrate a hydrogel-loading-dependent release of Raman-detectable hydrogel-associated material, while only minor differences were observed between washed and unwashed samples at the same hydrogel loading. However, while the Raman signal supports the release of carboxylate-containing hydrogel-derived species into the aqueous phase, it does not allow differentiation between monomeric, oligomeric, or polymeric species.

**Figure 10:**
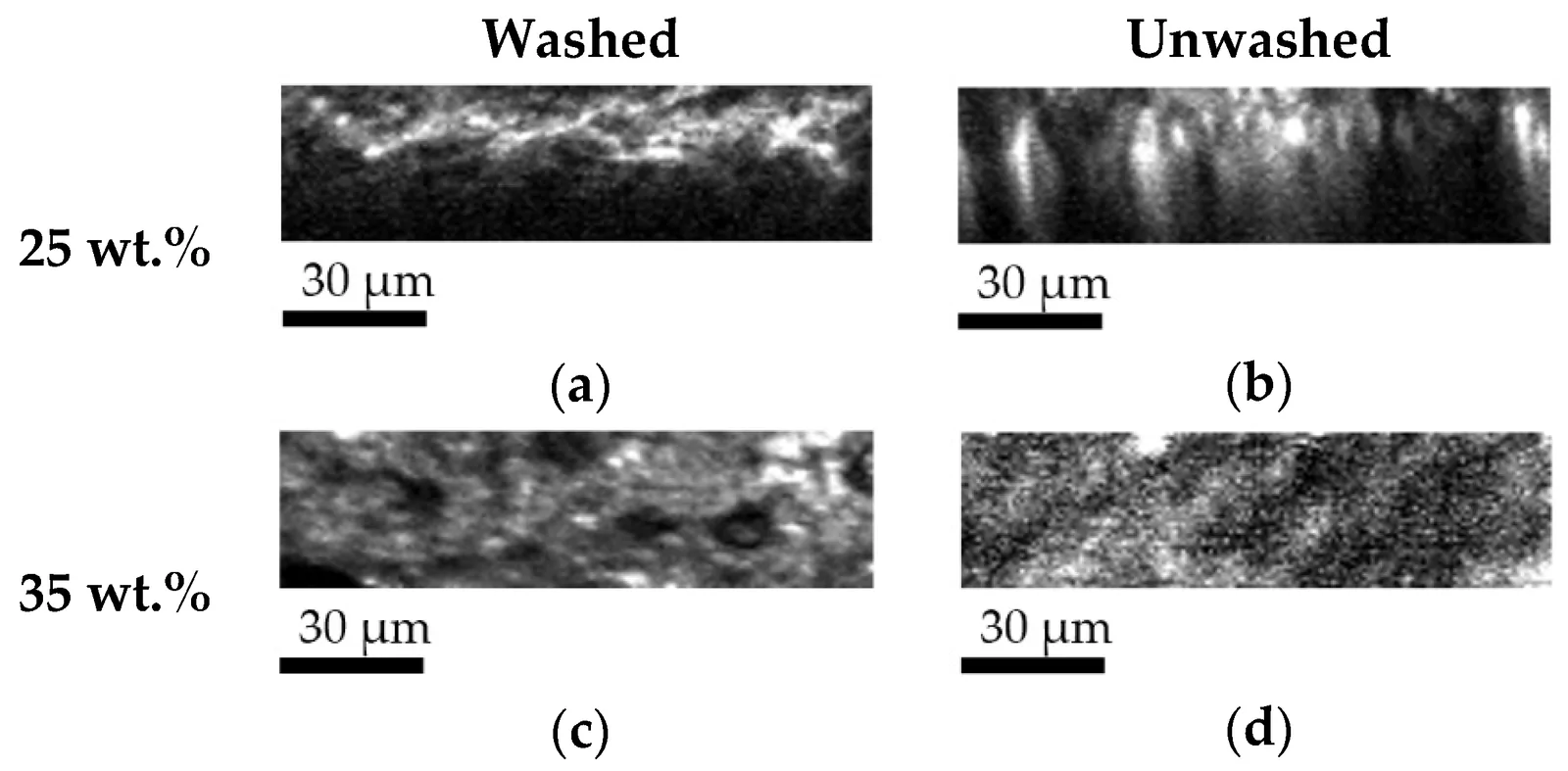
Comparative Raman maps of representative composite regions in swollen states, showing the PDMS-associated signals at 2908 cm^−1^. Changes in the spatial definition and local intensity of the PDMS-rich domains after swelling are shown. Maps are shown for the available 25 wt.% hydrogel formulation in washed (**a**)/unwashed (**b**) processing conditions and 35 wt.% hydrogel formulation in washed (**c**)/unwashed (**d**) processing conditions. The amide-associated regression intervals are shown in Appendix A, Figure A4.

To evaluate the microstructural changes induced by water uptake, comparative Raman imaging was performed on swollen composite specimens (Figure 10). In the dry state, distinct and spatially confined amide-rich domains were clearly recognizable via the characteristic band at 1617 cm^−1^. Following immersion and swelling, however, these amide-associated signals were substantially reduced and no longer clearly resolved as spatially confined domains.

### 3.4. In Vitro Cytotoxicity

**Figure 11:**
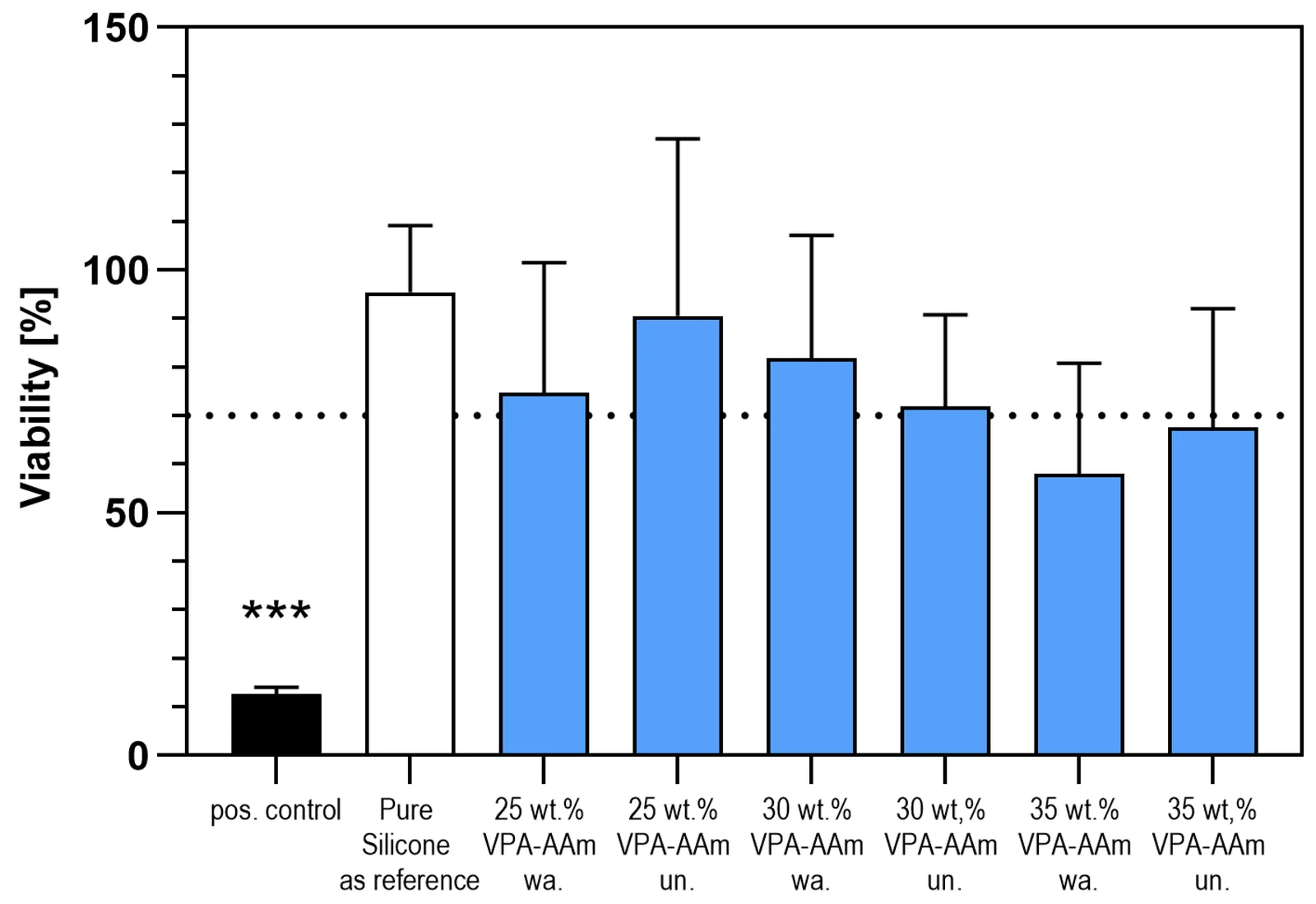
In vitro cell viability determined by WST-1 assay after exposure to extracts of pure silicone and VPA–AAm/silicone composites containing 25, 30, or 35 wt.% hydrogel, either washed (wa.) or unwashed (un.). Data are presented as mean ± SD from five replicates. The dotted horizontal line indicates the 70% cell-viability threshold used as a reference for cytotoxicity assessment. Statistical differences were evaluated by one-way ANOVA followed by Dunnett’s multiple-comparisons test against the blank extract (100% viability): *** *p* < 0.001.

The metabolic competence of cells exposed to the printed composite extracts was evaluated via a WST-1 assay as a measure for viability. The quantitative viability results relative to the blank extract are illustrated in Figure 11. According to ISO 10993-5 [22], a cell viability below the threshold of 70% (indicated by the dotted line) indicates a cytotoxic effect.

The pure silicone sample served as a negative reference material and showed a mean cell viability of approximately 95.4 ± 13.81%. Both the washed (wa.) and unwashed (un.) formulations at 25 wt.% hydrogel loading showed mean cell viabilities above the 70% threshold, reaching 75% ± 26.93% and 90.6 ± 36.43%, respectively. Likewise, both 30 wt.% formulations remained above the threshold, with mean viabilities of 81.8% ± 25.36% for the washed and 71.1% ± 18.81% for the unwashed formulation. At the highest filler loading of 35 wt.%, mean cell viability decreased below the 70% threshold for both formulations, reaching 58.0 ± 22.78% for the washed and 67.6% ± 24.49% for the unwashed samples.

One-way ANOVA revealed significant differences in cell viability among the investigated extract conditions (F(10,40) = 3.52, *p* = 0.002). Subsequent Dunnett’s multiple-comparisons test against the blank extract showed a significant reduction in cell viability for the positive control (adjusted *p* < 0.001). In contrast, none of the six VPA–AAm/silicone composite extracts differed significantly from the blank extract (all adjusted *p* > 0.05).

## 4. Discussion

### 4.1. Hydrogel Processing

The fine milling and sieving of the VPA-AAm hydrogel to a target size of 20–50 µm highlighted the challenges associated with processing highly elastic and cohesive polymer networks. Repeated milling was required to obtain the desired particle fraction. A crucial improvement was achieved by pre-washing the hydrogel powder prior to final drying and sieving. This treatment likely removes low-molecular-weight synthesis residues, unreacted synthesis precursors, or other soluble species from the particle surfaces, thereby reducing capillary adhesion and interparticle forces during subsequent drying. Consequently, particle agglomeration was reduced and sieving efficiency improved, providing a sufficient quantity of fine filler material for downstream 3D printing.

The morphological evaluation of the 3D-printed composites revealed two distinct phenomena: loss of transparency upon filler addition and the formation of micropores during thermal curing. Complete opacity was already observed at the lowest VPA-AAm loading of 25 wt.%, which can be attributed to the refractive index mismatch between the silicone matrix and embedded hydrogel particles and the resulting light scattering. No visually discernible differences in transparency or color were observed between the 25, 30, and 35 wt.% formulations.

Regarding the formation of internal voids, the experimental data indicate that the observed porosity is not primarily due to air entrapped during the mechanical mixing phase. Since all formulations were subjected to identical vacuum degassing procedures and the uncured reference sample remained completely defect-free, the pore formation is clearly linked to the rapid thermal curing cycle at 150 °C. The slight increase in bubble density at higher hydrogel concentrations suggests that the hydrogel phase—due to its highly hydrophilic properties—retains molecular traces of residual moisture despite the drying process. Upon sudden exposure to 150 °C, this bound water vaporizes rapidly. Because the addition-curing reaction of the silicone matrix progresses extremely fast at this elevated temperature, the expanding water vapor becomes trapped within the rapidly crosslinking, viscous network before it can diffuse to the sample surface and escape. The fact that prior vacuum degassing failed to eliminate these voids confirms that they represent a secondary product of the thermal expansion of moisture trapped within the VPA-AAm domains during the curing process, potentially exacerbated by an insufficient degassing time relative to the increased viscosity of the highly filled composites.

### 4.2. Fluorescent Labeling and Percolation Behavior

The successful labeling of the VPA-AAm hydrogel with Rhodamine B provided an intense fluorescence signal and a sharp contrast against the non-fluorescent silicone matrix, allowing for a precise evaluation of the internal phase morphology. The key structural insight gained from fluorescence microscopy is the absence of an apparent continuous or percolating hydrogel network in the investigated regions. Instead, the hydrogel phase was observed as isolated, well-dispersed domains, confirming that the predefined particle size distribution (20–50 µm) was successfully maintained both during the printing process and the subsequent thermal curing. This morphology may be attributed to efficient wetting of the hydrogel particles by the silicone matrix, maintaining a continuous silicone phase between neighboring particles, meaning that a substantially higher volume fraction would be required to enforce a continuous, interconnected framework.

The continuous silicone phase surrounding the isolated hydrogel domains is advantageous for maintaining the elastomeric character of the composite, which is particularly relevant for flexible electrode carriers. The silicone matrix used in this study (NuSil MED-4850) belongs to the manufacturer’s implant-line materials and was selected to maintain compatibility with established silicone-based implant concepts. Importantly, retaining a silicone-based carrier matrix avoids introducing an entirely new structural carrier material, while the hydrogel phase provides the additional swelling functionality. This may facilitate the future integration of the actuator concept into existing silicone-based implant architectures by restricting the material modification to selected functional regions rather than requiring replacement of the complete carrier system. In this context, extrusion-based additive manufacturing may provide a particularly suitable route for locally depositing the hydrogel–silicone composite as an additional actuator layer or functional region onto an existing silicone-based structure. Nevertheless, the established use of the silicone itself does not establish the biological suitability of the resulting hydrogel–silicone composite. In particular, potential low-molecular-weight extractables from the composite and their long-term release under physiological conditions require further investigation before chronic implantation can be considered. Moreover, since each hydrogel domain is fully encapsulated by the surrounding silicone, the particles are mechanically locked within the matrix. The isolated morphology also requires water and other molecules to pass through the surrounding silicone rather than through continuous hydrogel channels, resulting in diffusion-controlled transport through the composite [23].

On the other hand, the lack of an interconnected channel network limits water transport into the composite core, as the hydrophobic silicone matrix acts as a continuous diffusion barrier. Consequently, hydrogel domains located deeper within the composite may participate in swelling only at later stages. As the isolated hydrogel domains absorb water and expand, they exert localized mechanical stresses on the surrounding silicone. At high filler loadings such as 35 wt.%, the reduced silicone thickness between neighboring domains may promote microcracking or microvoid formation and thereby facilitate long-term hydrogel loss from the composite [18].

### 4.3. Swelling Behavior and Gravimetric Kinetics

The gravimetric swelling curves of the 3D-printed composites exhibit a distinct biphasic kinetic profile, which provides insights into the underlying mass transport mechanisms. The initial, rapid increase in the swelling rate within the first eight hours reflects a predominantly diffusion-controlled mechanism governed by non-Fickian transport kinetics. This behavior is consistent with findings by Bian et al., who demonstrated that despite its macroscopic hydrophobicity, PDMS exhibits an anomalous water vapor permeability driven by molecular transport through free-volume nanostructures [24]. Here, the Raman maps support a particulate composite architecture in which hydrogel-rich domains are embedded within a continuous PDMS phase. Accordingly, water uptake does not require a pre-existing percolating hydrogel network, because water can reach isolated hydrogel domains by transport through the surrounding silicone matrix. The observed micropores may locally modify these transport pathways by reducing the effective silicone thickness between adjacent hydrogel particles and the surrounding medium, potentially facilitating water uptake. Their quantitative contribution to swelling kinetics and potential effects on mechanical or long-term stability cannot be determined from the present data. Increasing the hydrogel loading reduces the characteristic amount and distance of PDMS separating neighboring hydrogel domains, which facilitates water access to the dispersed hydrogel phase.

As quantified by the initial apparent diffusion coefficients (*D_app_*), this transport is accelerated in the 35 wt.% formulations, where the higher particle density provides much shorter inter-particle distances. However, the swelling trends invert as the immersion time approaches equilibrium. Between 24 and 48 h, the formulations transition toward a plateau, where hydrogel water uptake is increasingly balanced by the elastic restrictive forces of the silicone matrix.

The occurrence of the absolute maximum swelling capacity at the intermediate filler loading of 30 wt.% (up to 460% for the unwashed state), rather than at the highest loading of 35 wt.%, can be explained by the complex interplay between osmotic pressure and the mechanical expansion resistance of the matrix. At a filler concentration of 30 wt.%, an optimal compromise is achieved between a sufficiently high density of hydrophilic hydrogel domains and the remaining elastomeric silicone walls. In this state, the hydrogel can generate sufficient osmotic pressure to elastically stretch the surrounding silicone without prematurely overstraining the elastic recovery capacity of the matrix.

Conversely, increasing the filler content to 35 wt.% leads to a drastic decline in long-term swelling performance, characterized by a gradual downward trend after its peak at 7 h, decreasing from approximately 330% to 290% after 4 weeks. At this maximum loading, the densely packed particles leave only thin silicone walls that may be further compromised by thermally induced microvoids. Swelling of the hydrogel domains may therefore generate high local mechanical stresses, promoting microcracking at the silicone–particle interfaces and compromising the sealing effect of the elastomeric matrix. Consequently, rather than true hydrogel shrinkage, the 35 wt.% formulations may undergo a permanent loss of hydrogel from the composite into the water medium over the course of weeks. While the internal hydrogel domains remain fully hydrated, this steady net mass loss reduces the dry weight of the composite over time. In the gravimetric analysis, this progressive mass loss manifests as an apparent decrease in the calculated swelling ratio during long-term measurements, although the remaining hydrogel domains may remain hydrated.

Consistent with these observations, post-swelling Raman mapping showed a pronounced reduction in the intensity and spatial definition of the amide-associated signal. However, this reduction cannot be interpreted as standalone evidence for hydrogel loss. As the embedded hydrogel domains absorb water and expand, the amide-containing polymer is distributed over a larger hydrated volume, reducing its local concentration and thus the Raman contrast within the mapped volume. The presence of absorbed water may additionally affect the local measurement environment. Consequently, the reduced amide-associated Raman signal after swelling may result from swelling-induced dilution and redistribution of the hydrogel phase, while partial material release may contribute additionally.

Altogether, these structural changes and network expansion directly influence the elution kinetics of entrapped chemical species, including potentially extractable or leachable compounds, consistent with transport-controlled release mechanisms described for smart hydrogel systems [16,25,26,27].

The influence of the purification state (unwashed vs. washed) observed for the 30 wt.% and 35 wt.% formulations in the long-term curves may be related to soluble hydrogel-associated species, synthesis residues, or ionic species remaining in the unwashed VPA-AAm phase. While the washing process removes these residues and creates microstructural voids that accelerate the early-stage diffusion velocity (*D_app_*), the unwashed formulations benefit from a different thermodynamic driver in the long run. These species may increase the ionic concentration within the particles and thereby generate a higher osmotic pressure gradient relative to the surrounding medium, promoting greater long-term water uptake than in the washed counterparts over hundreds of hours. The disappearance of this effect after 24 h at 25 wt.%, where both curves converge, indicates that at lower particle densities the constraining effect of the silicone matrix may dominate over these additional osmotic contributions [16,28].

However, when considering these composites for long-term implantable devices—such as flexible electrode carriers—the strict operational requirements demand structural stability far beyond the timeframe evaluated here. While the 28-day swelling duration in this study provides valuable fundamental insights into the initial hydration kinetics, network capacity, and early elution behavior, it remains inadequate for fully predicting multi-year in vivo performance. To address these temporal limitations, future research must extend toward long-term degradation, swelling and leaching studies over several months, accompanied by extended biological evaluation and accelerated-aging tests to investigate potential long-term fatigue, interfacial integrity, and mechanical endurance under physiological conditions.

### 4.4. Cell Viability

For the hydrogel-containing composites, the concentration-dependent behavior represents a crucial finding. Swelling of the embedded VPA-AAm particles facilitates bidirectional mass transport through the surrounding silicone matrix, with water entering the composite while potentially cytotoxic components are released.

The 25 wt.% and 30 wt.% formulations successfully exceed the cytotoxicity threshold, reaching viabilities of up to 91%. However, the 35 wt.% formulation did not pass this threshold, indicating an in vitro cytotoxic response [22].

The washed and unwashed formulations showed differences in mean cell viability; however, no consistent effect of the washing treatment was observed across the investigated hydrogel loadings. Although the Raman signal of released hydrogel-associated species increased with hydrogel loading, washed and unwashed samples at the same loading exhibited only minor differences in the evaluated Raman-band amplitude. Thus, the differences in biological response cannot simply be attributed to differences in the amount of Raman-detectable hydrogel-associated material but may instead be related to the hydrogel processing sequence.

The hydrogel was initially purified by repeated solvent exchange with isopropanol after polymerization. Nevertheless, residual low-molecular-weight synthesis-related species cannot be excluded by the present analyses, and the subsequent ethanol washing should therefore be regarded as an additional post-treatment rather than the sole purification step.

A possible explanation lies in the processing sequence. In the present procedure, ethanol washing preceded final milling to 20–50 µm. While washing removes readily accessible soluble species from the existing particle surfaces, subsequent milling creates fresh fracture surfaces and exposes previously inaccessible internal regions. In addition, washing and drying may alter the internal particle morphology; increased porosity could enhance solvent accessibility and mass transport [29] while also promoting fragmentation during subsequent milling and thereby exposing additional low-molecular-weight or soluble species. Together, these effects provide a plausible explanation for why the washed formulations did not consistently exhibit higher cell viability.

Consequently, performing a final extraction after milling to the target particle size may improve purification by increasing the fraction of the final particle surface directly accessible to the extraction medium, thereby enhancing the removal of soluble hydrogel-associated species prior to incorporation into the silicone matrix [30].

The biological impact of this hydrogel-derived elution is clearly illustrated at the maximum filler loading of 35 wt.%, where the extracts exhibit biologically relevant cytotoxicity. This decline follows a classic concentration-dependent trend: the excessively high particle density and the geometrically thinned matrix walls accelerate the transport kinetics of soluble extractables. This leads to a rapid accumulation of released amide-containing hydrogel-associated species in the surrounding medium, reaching concentrations that impair cellular viability [26].

Ultimately, this underscores that the 30 wt.% formulation represents the optimal compromise: it limits the diffusive release of elutable species to such an extent that robust cell viability is maintained, while simultaneously preserving the physical integrity of the matrix.

While adjusting the filler fraction mitigates toxicity at the formulation level, the underlying issue of unpurified fracture surfaces remains. Thus, although ethanol washing itself may represent an effective purification step, the subsequent processing route should be reconsidered to preserve its effect throughout the preparation of the hydrogel–silicone bimorph.

Future work should therefore focus on both processing optimization and application-oriented evaluation. Alternative processing routes based on solvent exchange are currently being explored to improve hydrogel drying while minimizing the need for extensive post-drying milling. These include solvent exchange to tert-butanol, currently under investigation as a preparatory step for freeze-drying, as well as supercritical CO_2_-based processing. Both approaches may help preserve the hydrogel particle structure and reduce processing-induced fragmentation. Furthermore, dynamic perfusion-based testing could complement static extraction assays by evaluating release behavior and cellular responses under continuous medium exchange. Ultimately, in vivo studies will be required to assess local tissue responses, inflammatory reactions, structural integrity, and whether the intended swelling-induced actuation is maintained under physiological conditions [31,32,33].

In particular, solvent exchange with tert-butanol followed by freeze-drying is currently being investigated as a route to preserve separated hydrogel particles while reducing the need for subsequent mechanical comminution [34,35]. Direct incorporation from a tert-butanol-containing suspension could additionally be explored, provided that solvent compatibility with the silicone and complete solvent removal can be ensured [36]. Such a route could potentially circumvent both conventional drying and the subsequent milling step, thereby reducing particle re-agglomeration and the generation of fresh fracture surfaces. The feasibility of this approach, however, would depend strongly on solvent–silicone compatibility and the complete removal of tert-butanol during or after composite preparation.

A further approach could be the use of spray drying to obtain separated hydrogel microparticles directly from a suitable suspension. Spray-drying processes can facilitate the formation of discrete particles while limiting bulk agglomeration and may therefore provide a more controlled starting material for incorporation into the silicone matrix [37,38]. Depending on the achievable particle-size distribution and the influence of the drying conditions on hydrogel structure and swelling behavior, this could provide an alternative route toward a reproducible hydrogel powder without an additional milling step.

The correlation between the gravimetric swelling profiles and the cytotoxicity results provides further multiphysical understanding of how water uptake acts as a kinetic “gatekeeper” for mass transport and cell viability. Within these composite architectures, the hydrogel phase should not be viewed merely as a passive water reservoir, but rather as an active thermodynamic engine that regulates the elution of chemical species. During the rapid initial swelling phase observed within the first eight hours, the sudden influx of water molecules forces the hydration and subsequent volume expansion of the surface-accessible VPA-AAm domains. This expansion swells the network meshes, effectively opening the structural pathways of the surrounding silicone matrix and enabling a dual transport mechanism: water diffuses inward, while unbound matrix extractables and residual hydrogel species diffuse outward into the extraction medium [16,18].

Conversely, at 35 wt.% hydrogel loading, the high particle density and reduced silicone wall thickness may generate local mechanical stresses during swelling, promoting microcracking and compromising the barrier function of the matrix. While water can thus enter the composite more readily, these structural changes may conversely facilitate the release of hydrogel-associated species. This interpretation is consistent with the declining long-term swelling curve, which indicates progressive mass loss, and may contribute to the lower cell viability (58–68%) observed for the 35 wt.% extracts [4,17,39].

Overall, these findings highlight that the 30 wt.% formulation represents the optimal “sweet spot” among the investigated compositions, providing the most favorable balance between material performance and cell viability.

## 5. Conclusions

In this study, 3D-printed VPA-AAm hydrogel/silicone bimorphs were developed and evaluated as swellable materials for potential application in cochlear implant electrode carriers. Pre-washing of the hydrogel reduced particle agglomeration and facilitated preparation of the 20–50 µm filler fraction required for composite printing. However, the biological evaluation indicated that washing prior to fine milling may limit purification efficiency, as subsequent particle fracturing exposes previously inaccessible internal surfaces.

Morphological analyses showed spatially separated hydrogel-rich domains within a continuous silicone matrix. Among the investigated compositions, 30 wt.% hydrogel provided the most favorable swelling behavior, whereas increasing the loading to 35 wt.% was associated with reduced long-term swelling and indications of progressive material loss. In vitro WST-1 testing showed mean cell viabilities above the 70% threshold for the 25 and 30 wt.% formulations, whereas both 35 wt.% formulations failed to meet the 70% cell-viability criterion defined in ISO 10993-5 [22].

Overall, the 30 wt.% formulation represented the most favorable compromise between swelling performance, structural stability, and cell viability among the investigated compositions. Future work should focus on optimizing the purification and particle-processing sequence and on evaluating long-term structural and biological performance under physiologically relevant conditions.

## Figures and Tables

**Table 1:** Experimental design and sample overview of the fabricated VPA-AAm hydrogel/silicone composites, detailing hydrogel content and washing status.

Silicone Matrix	Hydrogel Content (wt.%)	Hydrogel Washing
NuSil MED-4850	25	Yes
		No
	30	Yes
		No
	35	Yes
		No

**Table 2:** Apparent diffusion coefficients (*D_app_*) of the 3D-printed composites calculated from early-stage swelling kinetics (0–8 h).

Formulation	Purification State	Apparent Diffusion Coefficient (*D_app_*) (cm^2^∙h^−1^) *
25 wt.% VPA-AAm	Washed	2.02 × 10ˉ^5^
	Unwashed	1.65 × 10ˉ^5^
30 wt.% VPA-AAm	Washed	6.44 × 10ˉ^5^
	Unwashed	2.15 × 10ˉ^5^
35 wt.% VPA-AAm	Washed	1.53 × 10ˉ^4^
	Unwashed	8.80 × 10ˉ^5^

* *D_app_* was derived using Fick’s short-term approximation based on an initial specimen half-thickness (h) of 0.05 cm. Detailed regression intervals for each formulation are provided in Appendix B (Table A1).

**Figure A1:**
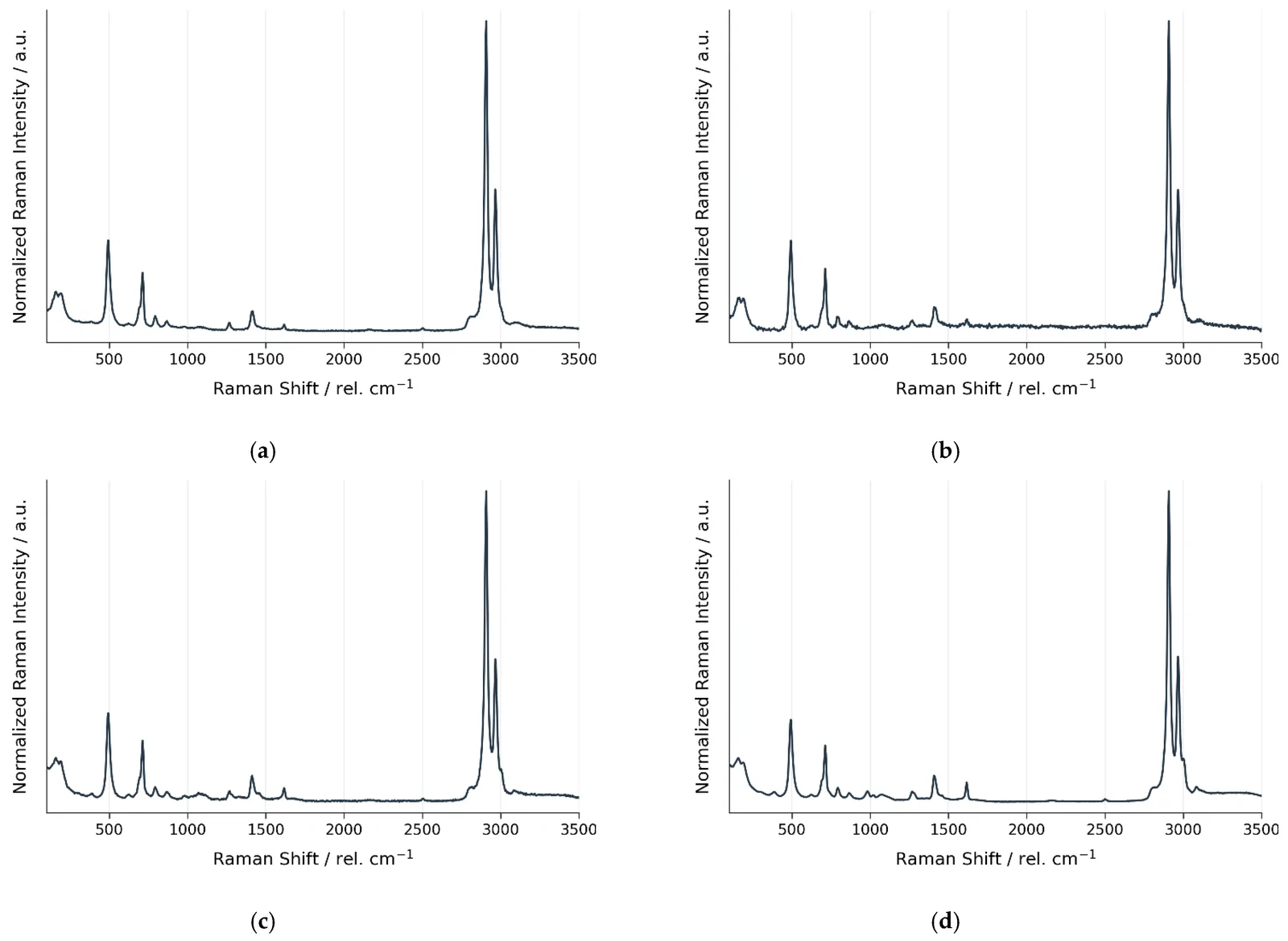
Normalized Raman spectra obtained from PDMS-rich regions of the investigated hydrogel–PDMS composites. Spectra are shown for the available 25 wt.% hydrogel formulation in washed (**a**)/unwashed (**b**) processing conditions and 35 wt.% hydrogel formulation in washed (**c**)/unwashed (**d**) processing conditions. The spectra document the bands used to assign the continuous PDMS-rich phase in the Raman imaging analysis.

**Figure A2:**
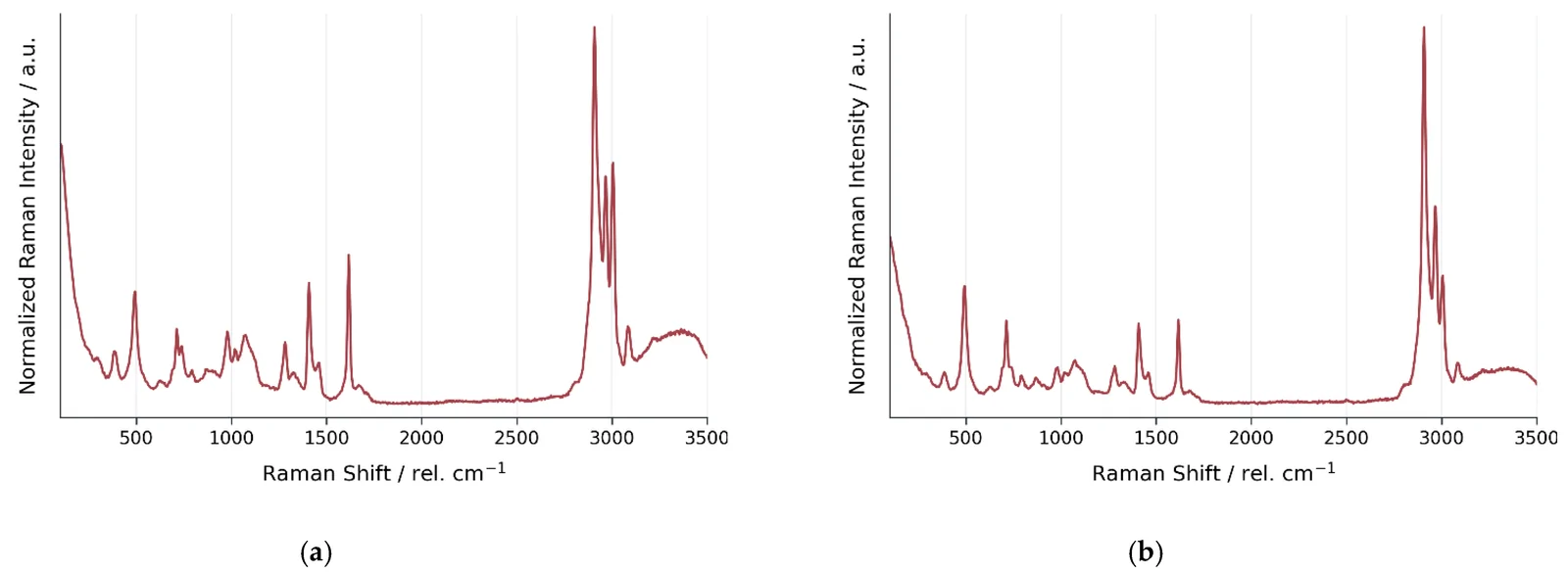

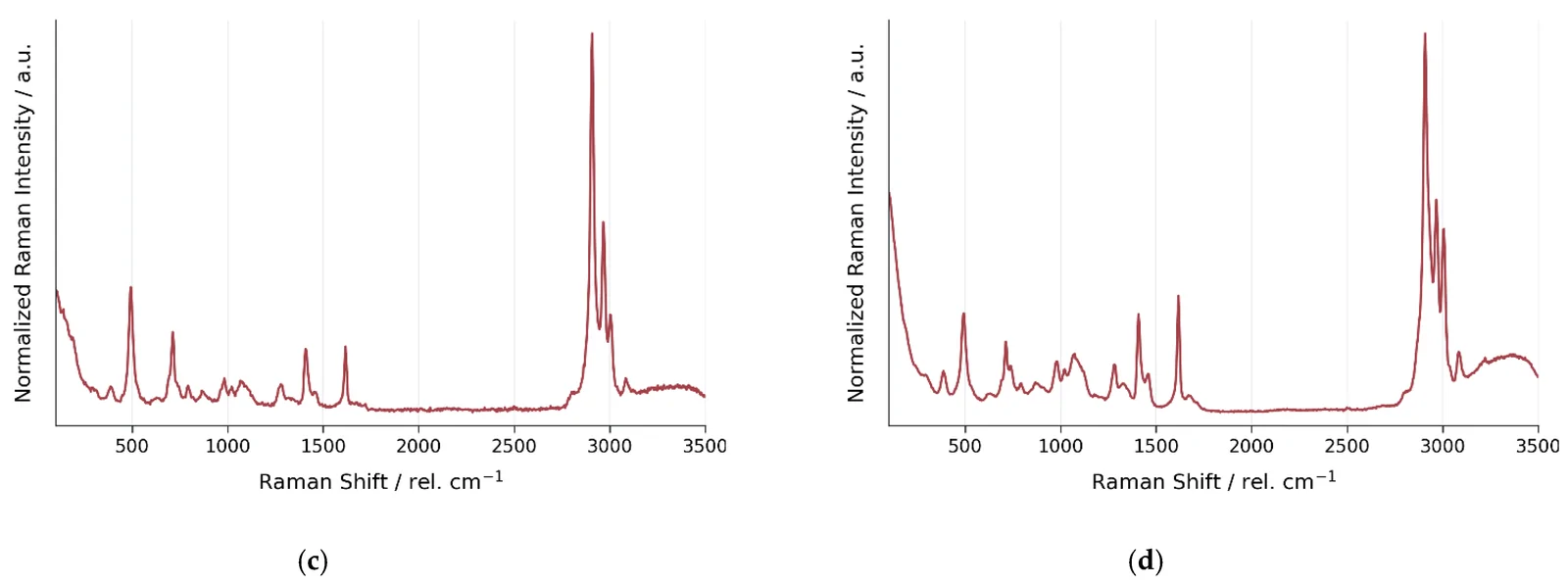
Normalized Raman spectra obtained from amide-rich regions assigned to the dispersed hydrogel phase. Spectra are shown for the available 25 wt.% hydrogel formulation in washed (**a**)/unwashed (**b**) processing conditions and 35 wt.% hydrogel formulation in washed (**c**)/unwashed (**d**) processing conditions. The amide-associated spectral features provide the basis for chemical identification of hydrogel-rich domains in the Raman maps.

**Figure A3:**
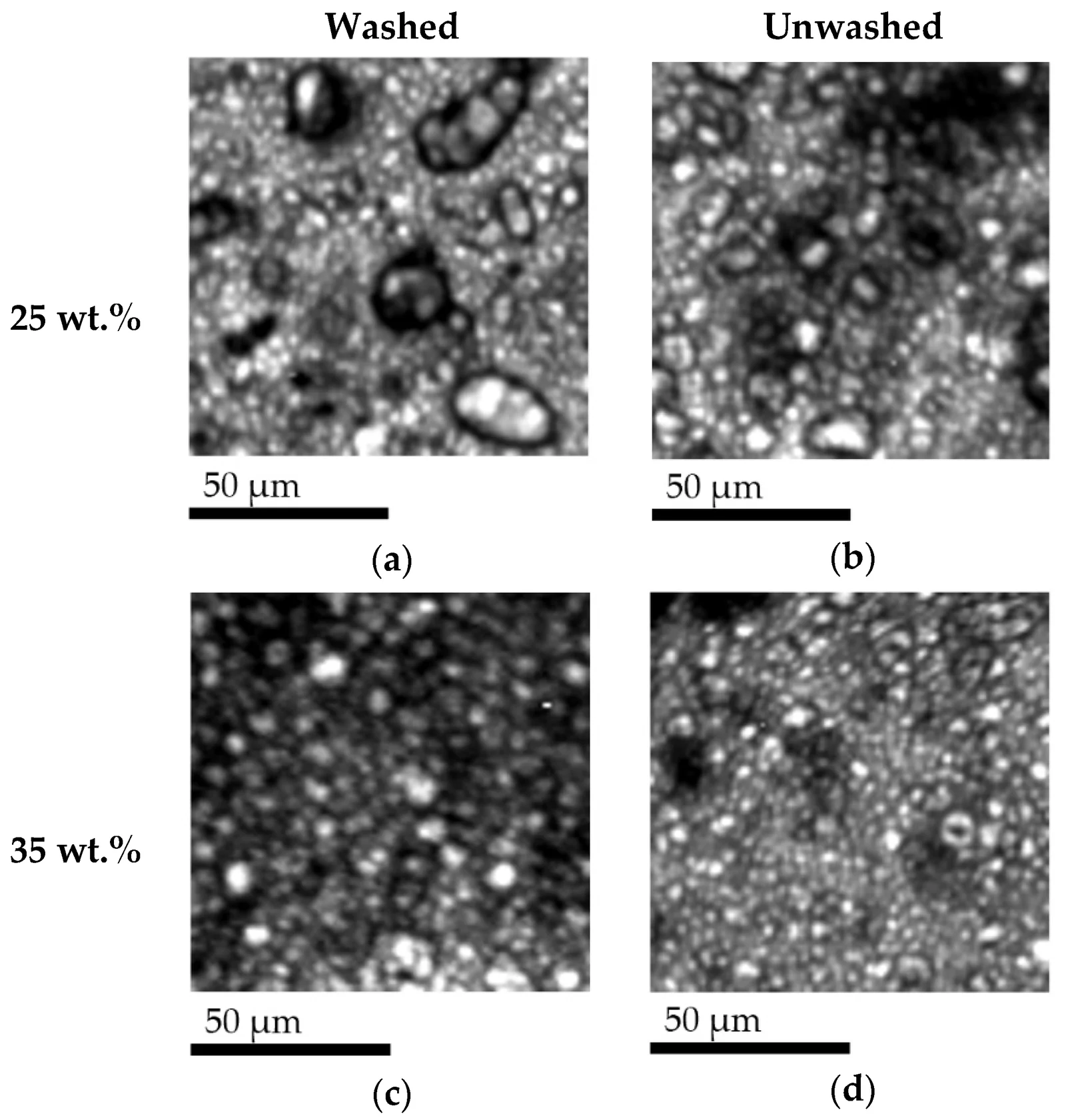
Raman imaging of PDMS-rich regions in unswollen states using the PDMS-associated band at 2908 cm^−1^ to distinguish the continuous silicone matrix from spatially confined hydrogel-rich domains. Maps are shown for the available 25 wt.% hydrogel formulation in washed (**a**)/unwashed (**b**) processing conditions and 35 wt.% hydrogel formulation in washed (**c**)/unwashed (**d**) processing conditions.

**Figure A4:**
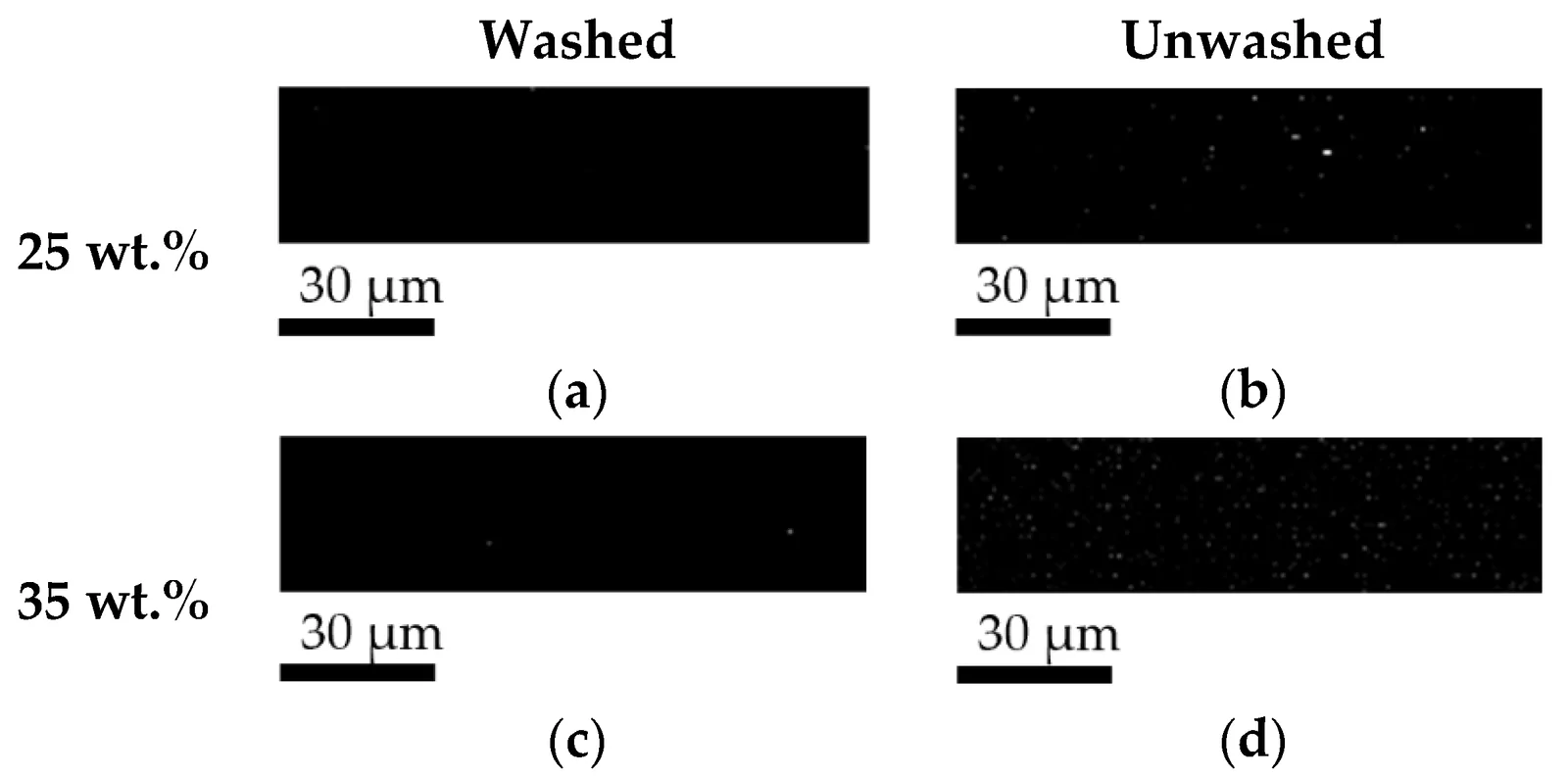
Comparative Raman maps of representative composite regions in swollen states, showing the amide-associated signals at 1617 cm^−1^. Changes in the spatial definition and local intensity of the amide-rich domains after swelling are shown. Maps are shown for the available 25 wt.% hydrogel formulation in washed (**a**)/unwashed (**b**) processing conditions and 35 wt.% hydrogel formulation in washed (**c**)/unwashed (**d**) processing conditions.

## Data Availability

The data presented in this study are available on request from the corresponding authors.

## Supplementary Materials

The following supporting information can be downloaded at: https://www.mdpi.com/article/10.3390/polym18192437/s1. S1. Preparation of the VPA–AAm Hydrogel.

## Abbreviations

The following abbreviations are used in this manuscript:

VPA	Vinylphosphonic acid
AAm	Acrylamide
MBAM	Methylenebisacrylamide
VPA-AAm	Vinylphosphonic acid–acrylamide
DMEM	Dulbecco’s Modified Eagle Medium
DMSO	Dimethyl sulfoxide
EDTA	Ethylenediaminetetraacetic acid
FBS	Fetal Bovine Serum
HBSS	Hanks’ Balanced Salt Solution
PBS	Phosphate-Buffered Saline
WST-1	Water-Soluble Tetrazolium-1 Assay
wa.	washed
un.	unwashed

## Appendix A

Complete single-point Raman spectra documenting baseline chemical assignments (amide band at 1617 cm^−1^ and PDMS C–H stretching at 2908 cm^−1^) across all composite formulations, alongside extended 2D Raman mapping datasets for the 25 wt.% and 35 wt.% (washed vs. unwashed) series, are provided (Figure A1, Figure A2 and Figure A3) to support the quantitative mapping and leaching analyses presented in the main manuscript.

## Appendix B

To provide a deeper insight into the early-stage transport kinetics, the initial swelling data (*M_t_*/*M_∞_* ≤ 0.6) were mathematically evaluated to determine the apparent diffusion coefficient (*D_app_*), as compiled in Table A1.

**Table A1:** Linear regression parameters, slope (*k*), and calculated apparent diffusion coefficients (*D_app_*) derived from the initial swelling phase of the 3D-printed hydrogel/silicone composites in their washed and unwashed states.

Formulation	Purification State	Regression Linear Range ($\sqrt{t}$)	Slope (*k*)	Apparent Diffusion Coefficient (*D_app_*) (cm^2^∙h^−1^)
25 wt.% VPA-AAm	Washed	0–2.83	0.203	2.02 × 10^−5^
	Unwashed	0–2.83	0.183	1.65 × 10^−5^
30 wt.% VPA-AAm	Washed	0–1.41	0.362	6.44 × 10^−5^
	Unwashed	0–2.83	0.209	2.15 × 10^−5^
35 wt.% VPA-AAm	Washed	0–1.00	0.558	1.53 × 10^−4^
	Unwashed	0–1.41	0.424	8.80 × 10^−5^
