# Supplementary material for: Qualification of a Swellable Hydrogel–Silicone Bimorph for Implantable Actuation
Source: Polymers (Basel). 2026 Oct 7;18(19):2437. doi: 10.3390/polym18192437 (PMC13667694; doi:10.3390/polym18192437)
Supplement: Supplementary file 1 [file polymers-18-02437-s001.zip › polymers-4566687-supplementary.pdf]

Supplementary Information

Qualification of a Swellable Hydrogel–Silicone Bimorph for Implantable Actuation

Esma Dosdoğru, Adrian Onken, Sabine Hild, Patricia Torgau, David Schäffl, Jan Sündermann, Marc Müller, Johannes Aucktor, Hasso v. Zychlinski, and Theodor Doll

S1. Preparation of the VPA–AAm Hydrogel

A solution of sodium hydroxide (16.0 g, 400 mmol) in deionized water (0.10 L) was slowly added to a solution of vinylphosphonic acid (VPA; Thermo Scientific, 90 wt.% technical grade; 30.1 g, 251 mmol) in deionized water (0.35 L), adjusting the pH to 6.8. Acrylamide (AAm; Acros Organics, ≥99%, biochemical grade for electrophoresis; 17.8 g, 251 mmol) and N,N′-methylenebisacrylamide (MBAM; Acros Organics, ≥99%, biochemical grade for electrophoresis; 1.56 g, 10.1 mmol) were subsequently added at room temperature. After rinsing with an additional 50 mL of deionized water, the stirred mixture was purged with a gentle stream of argon for 1 h, yielding a clear solution.

Tetramethylethylenediamine (TMEDA; 0.15 mL, 1.00 mmol) and potassium peroxodisulfate (KPS; 2.0% w/v in water; 1.35 mL, 0.10 mmol) were then added to the stirred solution, while gentle argon bubbling was continued for another 1 h. The reaction mixture was subsequently maintained at ambient temperature for 22 h, after which a gel had formed.

The resulting hydrogel was divided into portions and immersed in 1.0 L of isopropanol. After 6 h, the supernatant was decanted and replaced with fresh isopropanol. This solvent exchange was repeated twice with 1.0 L of fresh isopropanol over a total period of 3 d. Finally, the wet solidified material was dried under reduced pressure at 35 °C overnight. The resulting brittle material was ground to a fine powder using a rotor mill, yielding approximately 55 g of dried hydrogel powder.
